# Stroma-associated FSTL3 is a factor of calcium channel-derived tumor fibrosis

**DOI:** 10.1038/s41598-023-48574-8

**Published:** 2023-12-03

**Authors:** Jie-pin Li, Yuan-jie Liu, Yi Yin, Ruo-nan Li, Wei Huang, Xi Zou

**Affiliations:** 1 Affiliated Hospital of Nanjing University of Chinese Medicine, Jiangsu Province Hospital of Chinese Medicine, Nanjing, 210029 Jiangsu China; 2 Key Laboratory of Tumor System Biology of Traditional Chinese Medicine, Nanjing, 210029 Jiangsu China; 3 No. 1 Clinical Medical College, Nanjing University of Chinese Medicine, Nanjing, 210023 Jiangsu China; 4 Shihezi Labor Personnel Dispute Arbitration Committee, Shihezi, 832000 China; 5 Jiangsu Collaborative Innovation Center of Traditional Chinese Medicine in Prevention and Treatment of Tumor, Nanjing, 210023 China

**Keywords:** Oncogenes, Tumour biomarkers, Tumour immunology

## Abstract

Hepatocellular carcinoma (HCC) is the most widespread histological form of primary liver cancer, and it faces great diagnostic and therapeutic difficulties owing to its tumor diversity. Herein, we aim to establish a unique prognostic molecular subtype (MST) and based on this to find potential therapeutic targets to develop new immunotherapeutic strategies. Using calcium channel molecules expression-based consensus clustering, we screened 371 HCC patients from The Cancer Genome Atlas to screen for possible MSTs. We distinguished core differential gene modules between varying MSTs, and Tumor Immune Dysfunction and Exclusion scores were employed for the reliable assessment of HCC patient immunotherapeutic response rate. Immunohistochemistry and Immunofluorescence staining were used for validation of predicted immunotherapy outcomes and underlying biological mechanisms, respectively. We identified two MSTs with different clinical characteristics and prognoses. Based on the significant differences between the two MSTs, we further identified Follistatin-like 3 (FSTL3) as a potential indicator of immunotherapy resistance and validated this result in our own cohort. Finally, we found that FSTL3 is predominantly expressed in HCC stromal components and that it is a factor in enhancing fibroblast-M2 macrophage signaling crosstalk, the function of which is relevant to the pathogenesis of HCC. The presence of two MSTs associated with the calcium channel phenotype in HCC patients may provide promising directions for overcoming immunotherapy resistance in HCC, and the promotion of FSTL3 expressed in stromal components for HCC hyperfibrosis may be responsible for the poor response rate to immunotherapy in Cluster 2 (C2) patients.

## Introduction

The 2020 World Health Organization’s International Agency for Research on Cancer report revealed that hepatocellular carcinoma (HCC) ranks 6th among malignant tumor incidence worldwide, and 3rd among cancer-related mortality^1^. In China, primary HCC is the 4th leading malignant tumor and the 2nd contributor to tumor-related mortality, imposing a heavy economic burden on the healthcare system^2,3^.

Cancer cells are known to have a potent immune editing capability and are central to the establishment of an immunosuppressive tumor microenvironment (TME)^4,5^. Monoclonal antibodies (mAb) that suppress PD-1/PD-L1 interactions by interacting with PD-1 or PD-L1 were shown to repair the immune response in the tumor microenvironment^6–8^. With the approval of checkpoint inhibitors that target PD-1/PD-L1 and CTLA-4 for HCC, tumor immunotherapy has revolutionized the treatment of HCC, and immunosuppressive TME is not only a target for immunotherapy, but also the basis for tumor immune evasion and acquired drug resistance after immunotherapy^9–11^.

Ca(2 +) signaling regulation is an important process for tumor growth^12^, including cell proliferation and apoptosis^13–15^, and there is a large body of research specifically addressing the Ca(2 +) signaling pathway in HCC^16,17^. The work of Mateus T Guerra et al. confirmed that re-expression of calcium channel proteins is the last common event in HCC^18^. There have been several sporadic studies suggesting a bidirectional regulation of calcium channel molecules in HCC TME^19,20^, however most of these works are limited to individual calcium channel proteins and do not allow a comprehensive understanding of their significance in the development of HCC^21,22^.

Herein, our analysis of The Cancer Genome Atlas (TCGA) whole transcriptome data recognized two prognosis-related molecular subtypes (MSTs) with distinct physiological profiles and clinical prognoses, which have also been validated in another independent cohort. Furthermore, based on differential genes between the two subtypes, an interplay network consisting of gene modules was constructed and from which the hub gene *FSTL3*, a gene not previously studied in HCC, was identified to be associated with immunosuppressive TME in this disease. We further investigated this gene and confirmed the association of calcium channel MSTs with HCC hyperfibrosis and with primary M2 macrophages by enrichment analysis and immune assessment algorithms. The simultaneous analyses of single cell (SC)-, IF-, and IHC-based studies strongly supported that the immunosuppressive function of *FSTL3* leads to difficulties in benefiting from immunotherapy in HCC patients. Thus, characterization of the immunosuppressive function of *FSTL3* based on the calcium channel phenotype may provide an effective immunotherapeutic strategy and help overcome the poor efficacy of immunotherapy in immune exclusion type patients.

## Materials and methods

### The study designs

**Figure 1:**
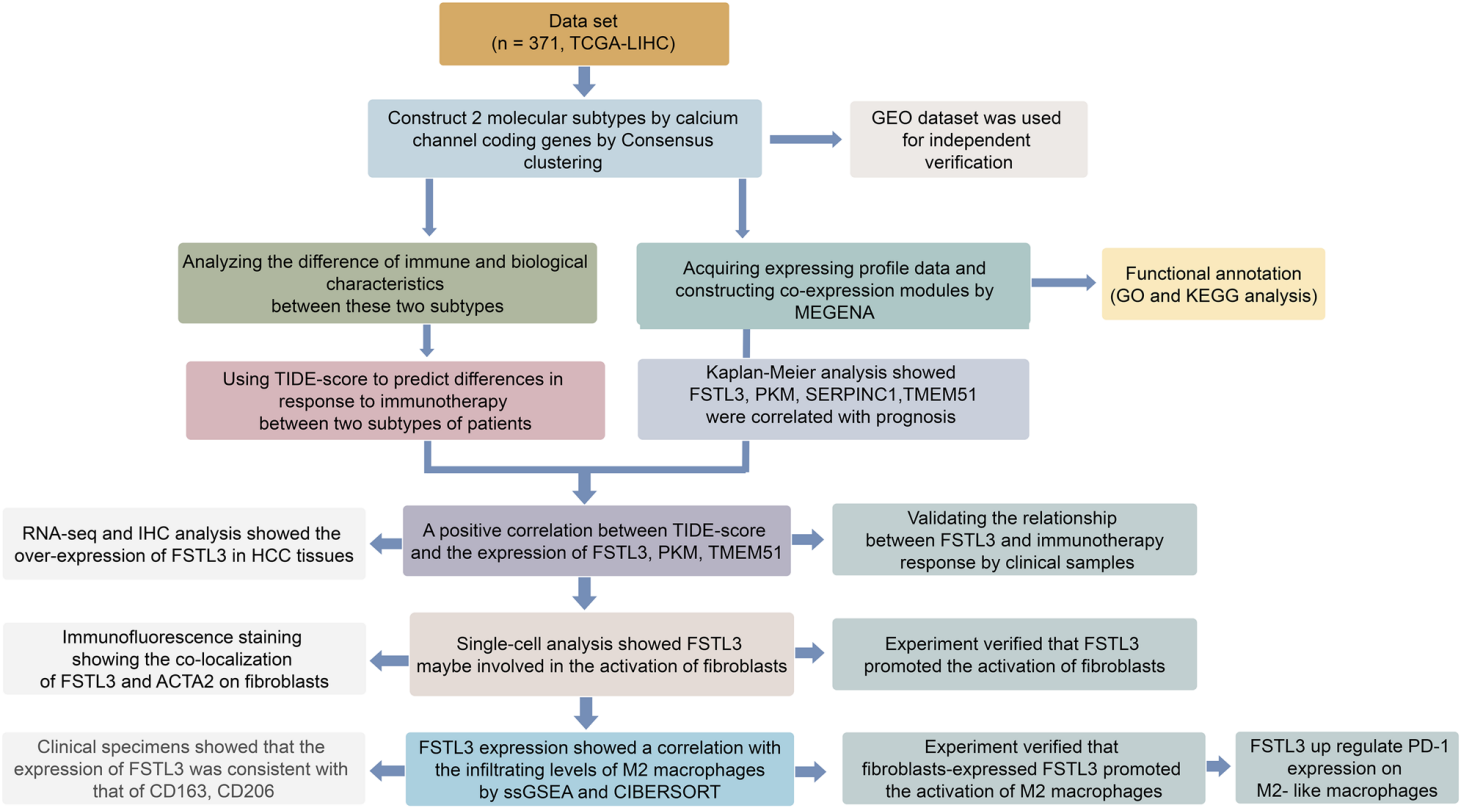
A schematic diagram of the research design.

Figure 1 illustrating our study design. In this study, we identified 2 distinct subtypes of HCC in TCGA cohort. Calcium channel subtypes were constructed by cluster analysis and Multiscale Embedded Gene co-expression Network Analysis (MEGENA) by using The TCGA and the Gene Expression Omnibus (GEO) database. Kaplan–Meier (K-M) curve described the prognosis in the different calcium channel molecules. The tumor immune dysfunction and exclusion algorithm was used to predict potential immune checkpoint inhibitors (ICI) therapy responses in different subtype. Single-cell RNA sequencing (scRNA-seq) and multiple bioinformatic/experimental approaches to analyze the role of FSTL3 in HCC.

### Antibodies, reagents, and cell lines

The details for the wet-lab experiments and all antibodies, reagents, and cell lines are summarized in Supplementary Materials.

### Ethics and sample collection

This research received ethical approval from Jiangsu Province Hospital of Chinese Medicine, Affiliated Hospital of Nanjing University of Chinese Medicine, Ethics and Research Committee (approval number: 2019NL-166-02), and strictly followed the Declaration of Helsinki^23^.

### Open access data source

We obtained information about 43 calcium channel-encoding genes from the HUGO Gene Nomenclature Committee (HGNC) portal^24^, as shown in Table S1. These genes were used for subsequent analysis.

In all, 371 HCC cases were recruited from TCGA^25^ through the University of California, Santa Cruz (UCSC) browser, and the data included genetic profiles [Fragments Per Kilobase Million (FPKM) values], corresponding clinical and mutational information^26^. The genetic demographics (FPKM values) of the TCGA-Liver HCC (LIHC) dataset^27^ underwent processing in R software for transformation to transcripts per kilobase million (TPM), which resemble microarray data. Owing to the lack of normal tissue samples in TCGA, we collected samples from Genotype-Tissue Expression (GTEx) database^28^ to serve as controls. Additionally, the Gene expression omnibus (GEO) dataset, particularly, GSE14520^29^, GSE36376^30^, GSE102079^31^, GSE10186^32^ was used for additional validation^33^. We also included a liver fibrosis dataset (GSE84044)^34^ to illustrate the relationship between calcium channel phenotypes and HCC background disease. Genetic profile retrieval utilized R (version 4.1.1) and data matrix construction was performed for subsequent analyses.

### Overview analysis of calcium channels encoding genes

The GENEMINIA database^35^ was used for annotation and clustering of calcium channel genes. The “corrplot” package was employed for association assessment between TCGA-LIHC-based calcium channel gene, and paired tumor-normal samples were used to compare differences in expression of calcium channel genes. Gene Set Cancer Analysis (GSCA) web tool^36^ analyzed the genetic profile, Copy-number variation (CNV), methylation, survival and function data of calcium channel genes in TCGA-LIHC. Finally, we visualized the mutation frequency of calcium channel genes in TCGA-LIHC using the “maftool” package.

### Consensus clustering for calcium channel molecules

We have provided a detailed list of the information on the 43 Calcium channel molecules from The Human Gene Nomenclature Committee (HGNC)^37^. Subsequently, the unsupervised clustering “Pam” method was employed using the “ConsensuClusterPlus” R package^38^, using the expression profile of the aforementioned 43 Calcium channel molecules, and the process was reiterated 1000 times to guarantee classification stability.

### Calcium channel phenotype-related DEGs

HCC patients were divided into different calcium channel clusters according to the expression profile of genes encoding calcium channel proteins to screen for calcium channel pattern-related genes. Differentially expressed genes (DEGs) between calcium channel subtypes were identified using criteria of *P* < 0.05 (after adjustment) and absolute fold change > 2 in the limma R package.

### Generation of co-expression axes

The “Multiscale Embedded Gene co-expression Network Analysis (MEGENA)” package in R software was employed for the screening of co-expression axes^39^. MEGENA is an innovative co-expression network analytical tool that provides multiple benefits over classical co-expression analytical approaches in effectively generating extensive co-expression plane filtering axes while maintaining gene–gene associations^39^. Fast Planar Filtered Network (PFN) generation is the initial stage of MEGENA analysis, followed by the computational acquirement of relevant gene pairs in PFN, and subsequent PFNs construction accumulated to Multiscale Clustering Analysis (MCA) for additional analyses^40^.

We retrieved the largest gene module from the co-expression axis, and transformed them to a readable format using cytoscape in order to perform our final analysis and visualization^41^. Lastly, we computed the degree values, which were necessary for the ranking of genes in the module to potentially uncover hub genes.

### Collection and processing of somatic alteration data

Associated mutational data for the TCGA-LIHC expression profile companion were retrieved from TCGA. This part of data was also employed to calculate the Tumor Mutational Burden (TMB)^42^ and Microsatellite Instability (MSI)^43^ of HCC. We identified the HCC driver genes by “maftool” R package and the leading 20 driver genes carrying the largest change frequencies were assessed in detail.

### Enrichment analysis

Gene Ontology (GO)^44^ and Kyoto Encyclopedia of Genes and Genomes (KEGG) enrichment analyses^45,46^ utilized the R “clusterProfiler” package. *P* < 0.05 was set as the significance threshold.

### Immune-related analysis

The various immune cell invasion status in HCC was quantified by using the “CIBERSORT” ^47^and “ssGSEA” R packages^48^, respectively, and the possible Immune Checkpoint Blockade (ICB) response of HCC patients was predicted by TIDE algorithm^49^. Immune and stromal scores were computed via the “ESTIMATE” package to estimate the content of invading immune and stromal cells in GSE36376 and GSE102079, and subsequently correlated immune and stromal fractions with FSTL3 levels by the spearman method.

### SC evaluation

Single-Cell RNA Sequencing (scRNA-seq) information was retrieved from https://www.ncbi.nlm.nih.gov/geo/query/acc.cgi?acc=GSE125449, and entered into Seurat V3. Filtered cells were visualized in Uniform Manifold Approximation and Projection (UMAP) and t-Distributed Stochastic Neighbor Embedding (t-SNE) following strict quality control raw Unique molecular identifier (UMI) > 200, mitochondrial gene percentage < 20%, log10 Gene per nUMI > 0.8)^50^. Subsequently, fibroblasts were identified using manual annotation. All cell type marker genes were identified using the “FindAllMarkers” function (min.pct = 0.25, logfc.threshold = 1, tes.use = ”wilcox”). “Dotplot” and “Vlnplot” functions plotted dot and violin plots, respectively. To further clarify the relationship of fibroblasts pseudotime trajectories with *FSTL3* and fibroblast activation markers, we conducted the Monocle 3 based on scRNA in HCC. We conducted the DDRTree method to reduce dimensionality and used the ‘plot_genes_in _pseudotime’ function to visualize the trend showing the dynamic profile of FSTL3 and fibroblast activation markers in the fibroblast pseudotime trajectories in HCC.

### Spatial transcriptomics

For the spatial transcriptome (ST) data, we obtained the hepatocellular carcinoma ST data from the GEO database (acquisition number: GSE203612)^51^. The “SpatialDimplot” function from the “Seurat” package was used to obtain the position information of each gene.

### Ethical approval and informed consent

The study was approved by Jiangsu Province Hospital of Chinese Medicine, Affiliated Hospital of Nanjing University of Chinese Medicine, Ethics and Research Committee (approval number: 2019NL-166-02). The study was in accordance with the declaration of Helsinki.

## Results

### An overview of the genetic characteristics, transcriptional variants and biological functions of 43 calcium channel molecules

**Figure 2:**
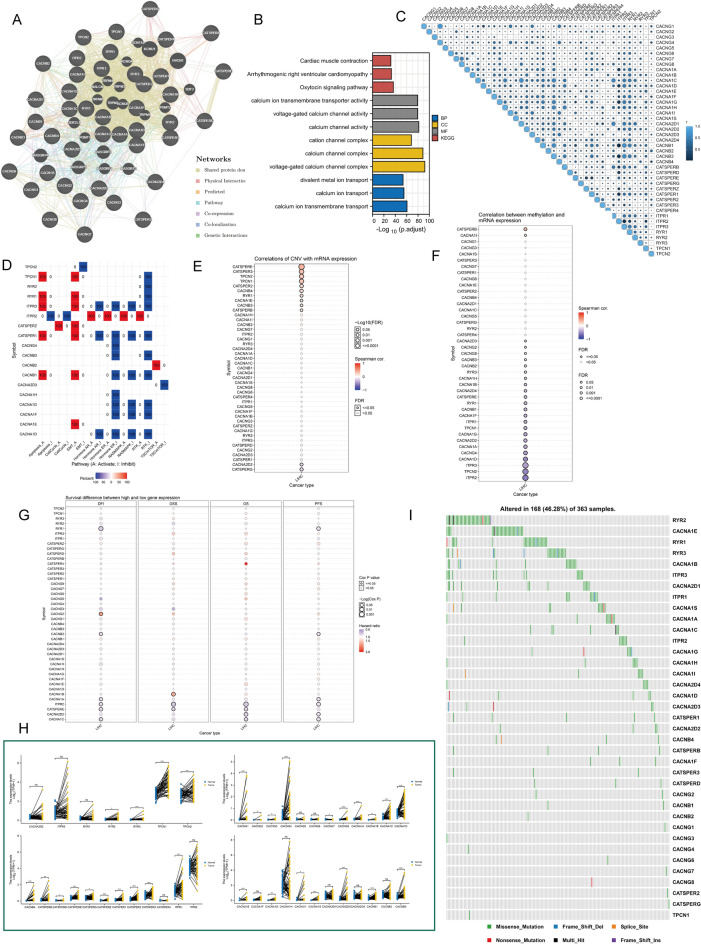
Expression variation of calcium channel molecules. (**A**) calcium channels with neighboring genes depicting physical associations, co-expression, co-localization, estimated common networks, genetic associations, and common protein domains. (**B**) The “clusterProfiler” R package was employed for the GO and KEGG enrichment analyses. Different colors represented different background genesets. (**C**) Interrelationship between the 43 calcium channel molecules. The bigger the size is and the lighter the color is, the higher the correlation is. (Spearman method, TCGA-LIHC, n = 370). (**D**) Heatmap depicting association between the 43 calcium channel molecule expressions in essential cancer-related networks. The global cancers percentage whereby a gene modulates the pathway in HCC, is shown as the percentage. “Pathway activate” (red) denotes cancers percentage whereby a signaling network may be potentially activated by specified genes, suppression depicted similarly as “pathway inhibit” (blue). (**E**) The CNV and mRNA expression correlation, as evidenced by the bubble chart. Red denotes positive association and blue negative association. A stronger color represents a larger association index. The bubble size represents the false discovery rate (FDR). (**F**) The 43 calcium channel molecule methylations and mRNA expression correlation, as evidenced by the bubble chart. Red denotes positive association whereas blue denotes negative association. A stronger color represents a larger association index. Bubble size represents the FDR. (**G**) The 43 calcium channel molecule mRNA expressions and HCC patients’ prognosis, as evidenced by the bubble chart. Red and blue denotes hazard ratio > 1 and < 1, respectively. A stronger darker color represents a larger association index. Bubble size represents −Log (Cox P). (**H**) Differential expression of 43 calcium channel molecules between HCC and matched normal tissues (Wilcoxon test, n = 50). (**I**) Mutation frequency of 43 calcium channel molecules in 363 HCC patients from the TCGA-LIHC dataset (n = 363).

We pooled data from extant studies for 43 calcium channel molecules, which are *CACNG1*, *CACNG2*, *CACNG3*, *CACNG4*, *CACNG5*, *CACNG6*, *CACNG7*, *CACNG8*, *CACNA1A*, *CACNA1B*, *CACNA1C*, *CACNA1D*, *CACNA1E*, *CACNA1F*, *CACNA1G*, *CACNA1H*, *CACNA1I*, *CACNA1S*, *CACNA2D1*, *CACNA2D2*, *CACNA2D3*, *CACNA2D4*, *CACNB1*, *CACNB2*, *CACNB3*, *CACNB4*, *CATSPERB*, *CATSPERD*, *CATSPERE*, *CATSPERG*, *CATSPERZ*, *CATSPER1*, *CATSPER2*, *CATSPER3*, *CATSPER4*, *ITPR1*, *ITPR2*, *ITPR3*, *RYR1*, *RYR2*, *RYR3*, *TPCN1*, *TPCN2*. Figure 2A demonstrated the network of calcium channel protein members and their interactive genes. Enrichment analysis revealed that calcium channel proteins are closely associated with myocardial composition and oxytocin signaling pathways, in addition to their involvement in conventional ion transport (Fig. 2B). Furthermore, we observed a strong co-expression association among 43 calcium channel molecules at the transcriptional level (Fig. 2C). We then examined the characteristics of the relevant networks. Most networks in HCC, particularly *ITPR3*, *CATSPER1*, and *CACNB1*, showed high levels of activation in the apoptotic and Epithelial-mesenchymal transition (EMT) signaling pathways, but consistent inhibition in the hormones Androgen receptor (AR), RAS/ Mitogen-activated protein kinases (MAPK), and Receptor tyrosine kinases (RTK) (Fig. 2D). We verified that genetic diversity critically modulated calcium channel molecule expressions. The CNV content and mRNA were directly associated in terms of a majority of calcium channel molecules, particularly in *CATSPERE* (Fig. 2E), while for methylation, gene methylation statuses were inversely associated with mRNA contents (Fig. 2F). Notably, the relationship between the levels of different calcium channel molecules and HCC patient prognoses was variable, with only elevated *CACNG2* and *CACNA1B* expressions being potential poor prognostic factors (Fig. 2G). Relative to the transcript contents of 43 calcium channel molecules in paired tissue samples, few genes were elevated in HCC tissues (Fig. 2H): *RYR2*, *RYR3*, *TPCN1*, *TPCN2*, *CACNB4*, *CATSPERB*, *CATSPERD*, *CATSPERE*, *CATSPERG*, *CATSPERZ*, *CATSPER1*, *CATSPER2*, *CATSPER3*, *ITPR1*, *CACNG1*, *CACNG2*, *CACNG4*, *CACNG8*, *CACNA1A*, *CACNA1B*, *CACNA1C*, *CACNA1D*, *CACNA1E*, *CACNA1G*, *CACNA1I*, *CACNA1S*, *CACNA2D3*, *CACNB1* and *CACNB3*. Among the 363 HCC samples, 168 had mutated calcium channel molecules with a frequency of 46.28%, with *RYR2* having the most mutational frequency, primarily missense mutation. (Fig. 2I). These results indicated strongly that aberrantly expressed calcium channel molecules in HCC was associated with cancer progression.

### Identification of 2 calcium channel subtypes based on consensus clustering

**Figure 3:**
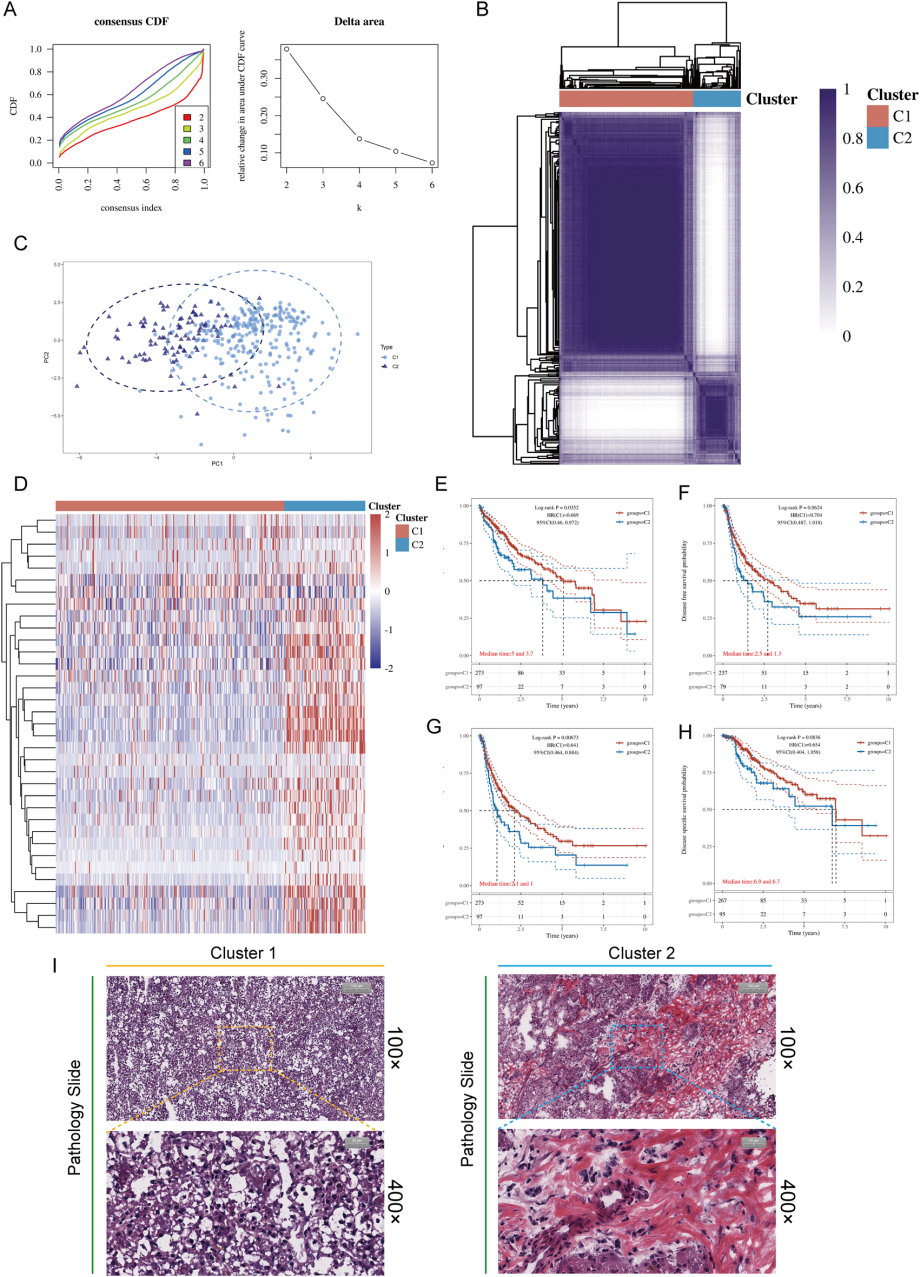
Unsupervised learning to identify 2 molecular subtypes (MSTs). (**A**) Left: The cumulative distribution function (CDF) curves of consensus scores via varying subtype numbers (k = 2, 3, 4, 5, and 6). Right: Relative area alteration under the CDF curve for k = 2–6. (**B**) The consensus score matrix of all samples when k = 2. A higher consensus score increased the potential of assignment to the same group. (**C**) The PCA distribution of TCGA-LIHC samples via calcium channel molecule expression profiles. Each point denotes a single sample; distinct colors denote the different subtypes. (**D**) Expressional distribution of 43 calcium channel molecules between the two subtypes. (**E**–**H**) Survival analysis including Overall Survival (OS) (**E**), progression-free survival (PFS) (**F**), disease-specific survival (DSS) (**G**), progression-free interval (PFI) (**H**) based on two subtypes (Logrank test, n = 370). (**I**) Typical images of pathological Hematoxylin and eosin (HE) staining of two calcium channel phenotypes.

To fully elucidate the integrated networks of calcium channels in HCC, we identified 2 unique pattern of calcium channel gene expression by unsupervised clustering, termed Cluster 1 (C1), Cluster 2 (C2) (Fig. 3A,B). Figure S1 depicts a suitable clustering effect when k = 2. GSE14520 was analyzed similar to TCGA-LIHC dataset (Figure S2). Based on PCA, the two clusters were effectively identified according to their expression profiles of 43 calcium channel molecules (Fig. 3C). The expression levels of calcium channels were then analyzed using heat maps, and we observed that calcium channel molecules were considerably upregulated in C2, compared to C1 (Fig. 3D). The Kaplan–Meier survival analysis of HCC patients demonstrated that patients in C2 have a relatively poor prognosis, including overall survival (OS) and disease-specific survival (DSS) (*P* < 0.05, Fig. 3E–H). Interestingly, according to the results of pathological tissue sections, we observed that the sample tissue of C2 had a more abundant collagen structure than that of C1 (Fig. 3I). These data suggested that variants in the expression of calcium channels may be associated with abnormal deposition of collagen. Considering that HCC develops as a consequence of chronic liver disease and that the vast majority of HCC occurs in patients suffering from liver fibrosis and cirrhosis, we further analyzed the correlation between this background disease and calcium channel phenotypes. We found that Scheuer staging fibrosis score (S0–S4) and Scheuer grading inflammation score (G0–G4) were higher in C2 patients compared to C1 in GSE84044 (Figure S3), suggesting that the patients with C2 features have more severe fibrosis even in the non-malignant stage.

### Clinical characteristics and biological functional differences associated with calcium channel phenotypes

**Figure 4:**
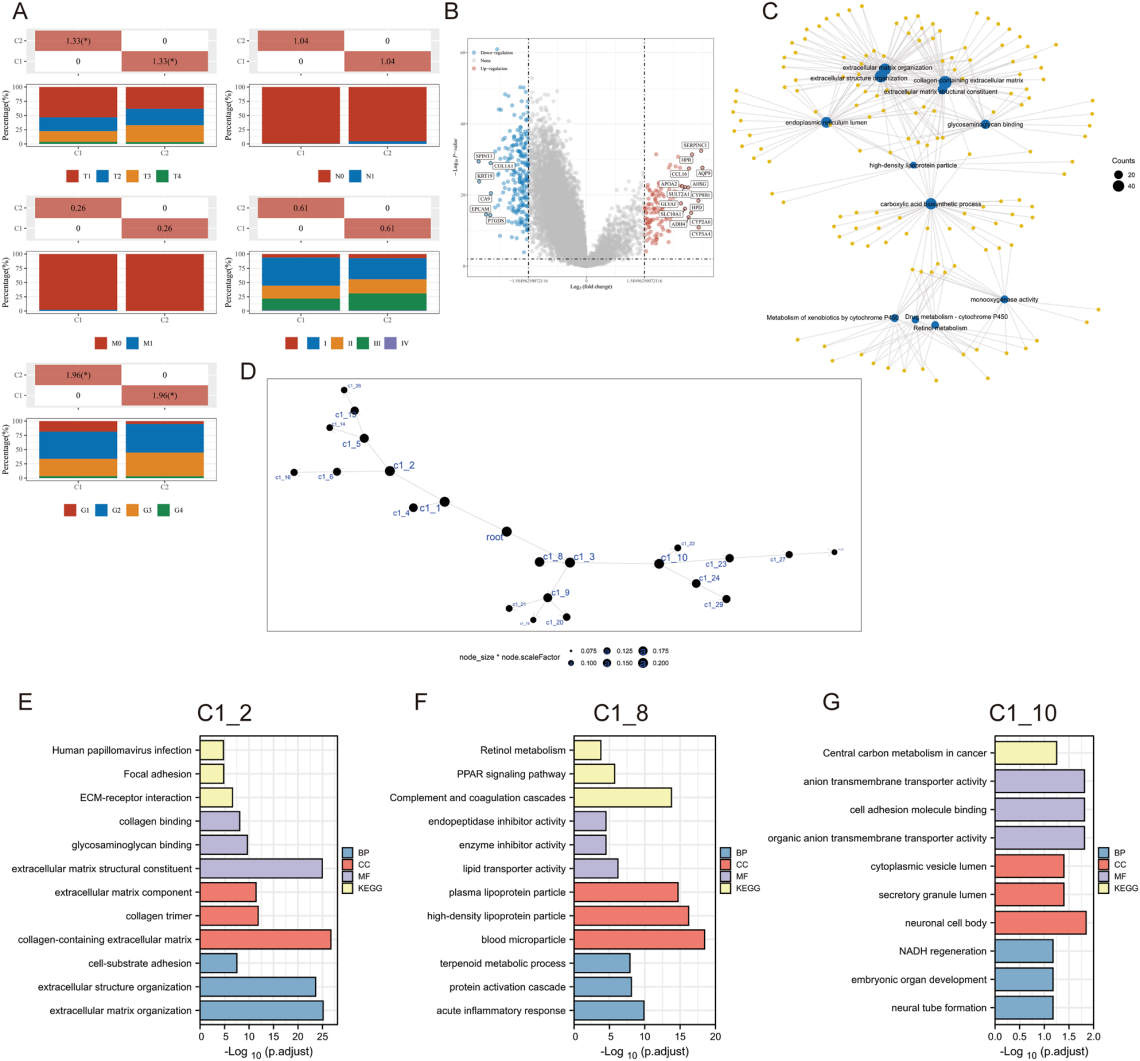
Analysis of calcium channel-related clinical characteristics and signal pathways differences. (**A**) Relationship between two subtypes and the clinicopathological parameters, including gender, T, N, and M stage, pathological stage, and tumor grade (*P < 0.05) (**B**) Expression difference analyses between the two subtypes were performed via the “limma” R package on TCGA-LIHC dataset, and a volcano plot was constructed. Blue, genes lowly expressed in C1; Red, genes highly expressed in C1; Grey, genes with no statistical difference in expression level. (**C**) The “clusterProfiler” R package was employed for GO enrichment analysis. (**D**) The co-expression network based on MEGENA analysis. Each node denotes a module, and larger nodes represent more quantity of genes. (**E**–**G**) GO and KEGG enrichment analyses of the 3 largest gene module, including C1_2 (**E**), C1_8 (**F**), and C1_10 (**G**). Each colored bar represents a distinct biological process. The vertical coordinate represents each term and the horizontal coordinate represents − Log10 (P.adjust).

We assessed alteration in the clinical profiles between the two groups at first and found that the type of C2 patients was more advanced compared to C1 (Fig. 4A). To further investigate the causes of the pathological differences between the two clusters, we obtained differential genes between the two clusters (Fig. 4B), and functional enrichment analysis (FEA) showed major enrichment in the extracellular matrix (ECM)-associated signaling pathways (Fig. 4C). In addition, in the cohort of Yujin Hoshida et al. we found that the majority of C1 were well-differentiated S3 subclass (good prognosis), and the majority of C2 were S1 and S2 subclasses at high risk of early recurrence (poor prognosis) (Figure S4A-B). Interestingly, TGFβ-activated signaling associated with an aggressive phenotype showed strong enrichment in S1; in our study, C2 showed more active EMT signaling (Figure S4C-D), which is associated with a tumor mesenchymal phenotype.

To screen for new modulatory targets between 2 clusters, we conducted the MEGENA algorithm to construct gene modules after aggregating all eligible DEGs (Fig. 4D). The largest module C1_2 showed 101 genes, C1_10 showed 89 genes and module C1_8 showed 74 genes (Figure S5A-C). FEA revealed that C1_2 was involved in ECM, and C1_8 was involved in high-density lipoprotein (Fig. 4E–G). Finally, we chose the three highest scoring genes in each module to conduct Kaplan–Meier plot and identified that *FSTL3* in C1_2, *PKM* and *TMEM51* in C1_8, and *SERPINC1* in C1 _10 were intricately linked to HCC patients’ outcome (*P* < 0.05, Figure S5D-F). Therefore, *FSTL3*, *PKM*, *TMEN51* and *SERPINC1* may be potentially valuable research targets.

### Identification of *FSTL3* as the main research objective

**Figure 5:**
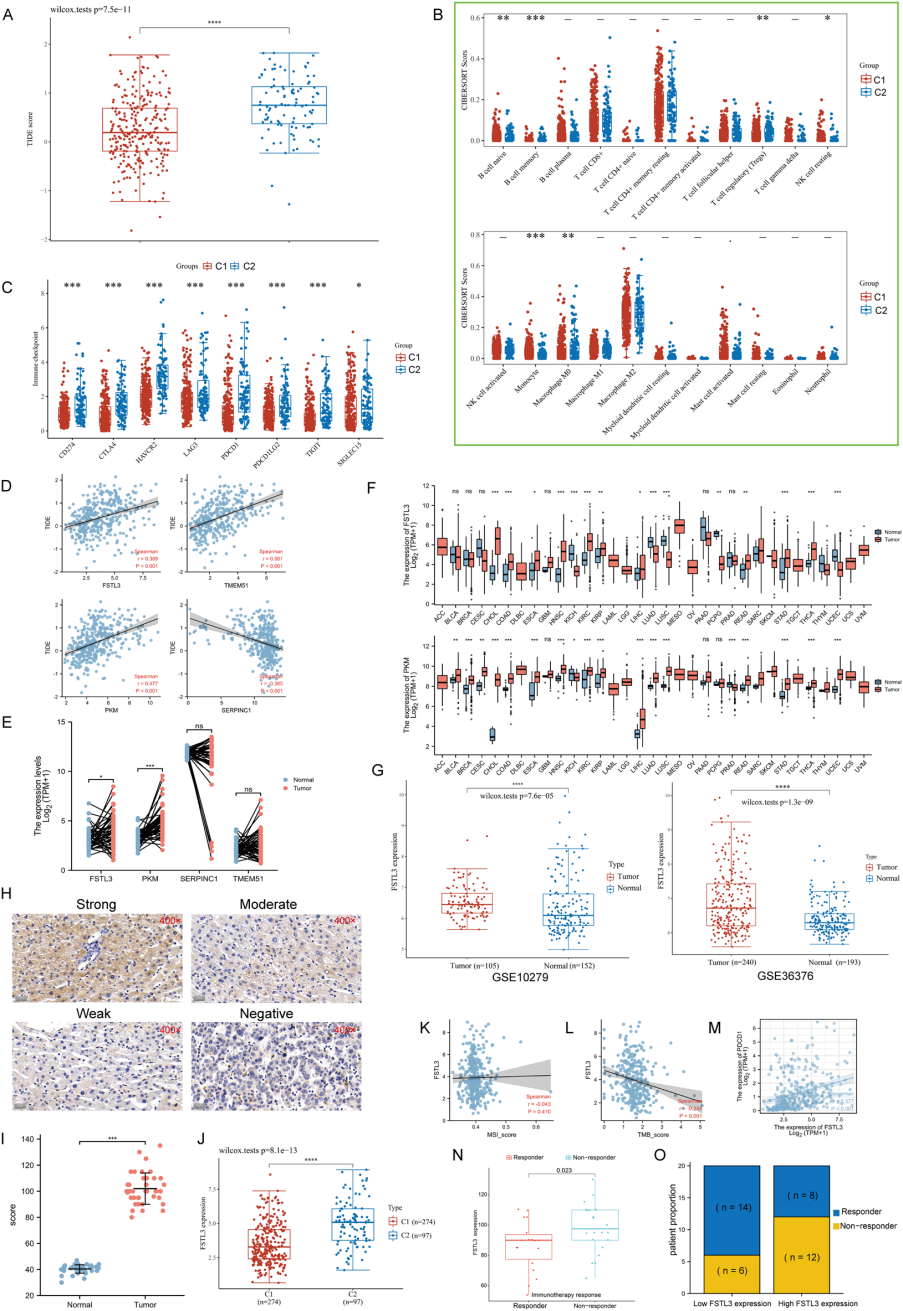
Analysis of the relationship between calcium channel phenotype and response to immunotherapy. (**A**) Analysis of TIDE score difference between the two subtypes (Wilcoxon test, TCGA-LIHC, n = 370). (**B**) Analysis of alterations in the immune cell abundance between two subtypes. The x-axis represents the immune cell type, and y-axis represents the immune score (CIBERSORT scores) in varying subtypes where distinct colors denote distinct subtypes. C1 (red) and C2 (blue), TCGA-LIHC. (**C**) Analysis of the differences in eight common ICPs between two subtypes (Wilcoxon test, TCGA-LIHC, n = 370). (**D**) Correlation between *FSTL3*, *TMEM51*, *PKM*, *SERPINC1* expression and TIDE score as evaluated based on TCGA-LIHC (Spearman method, n = 370). (**E**) Expression difference analyses of *FSTL3*, *TMEM51*, *PKM*, and *SERPINC1* based on TCGA-LIHC paired samples (Wilcoxon test, n = 50). (**F**) Human *FSTL3* and *PKM* expression levels in different tumor types from TCGA database. Wilcoxon test was conducted. (**G**) *FSTL3* expression differential analysis between tumor and normal tissues based on GSE10279 (left, n = 257), and GSE36376 (right, n = 433). Wilcoxon test was conducted. (**H**) Different IHC staining intensity of FSTL3 in HCC patients. (**I**) Statistical analysis of FSTL3 contents (H-SCORE) in normal and tumor tissues (Wilcoxon test, n = 40). (**J**) Expression difference analyses of *FSTL3* between C1 and C2 (Wilcoxon test, TCHA-LIHC, n = 50). (**K**–**M**) Correlation between *FSTL3* and MSI (K), TMB (L), as well as *PDCD1* (M), respectively (Spearman method, TCGA-LIHC, n = 370). (**N**) Box plot depicting different FSTL3 expressions between responder and non-responder following anti-PD-1 treatment in 40 HCC patients. (**O**) The high/low FSTL3 expression groups were divided depending on median of IHC scores of 40 HCC patients’ specimens. Bar plot depicting different response rates between elevated- and reduced-FSTL3 expression cohorts among 40 HCC patients. In the low FSTL3 expression groups, 14 patients were responders to anti-PD-1 therapy, and 6 patients were non-responders. There were 8 patients were responders to anti-PD-1 therapy, and 12 patients were non-responders in high FSTL3 expression groups. (**** *P* < 0.0001, *** *P* < 0.001, ** *P* < 0.01, * *P* < 0.05, ns not significant).

Considering that the desmoplastic reaction is the main trigger for resistance to anti-cancer immunotherapy, it is easy to understand that the significant difference in collagen connective tissue abundance between the two clusters will contribute to their different responses to immunotherapy^52–54^. Thus, we calculated TIDE score as an effective biomarker of ICB response, and we demonstrated that the TIDE score was substantially elevated in C2 than in C1, implying that patients in C2 had a relatively unfavorable response to immunotherapy (*P* < 0.001, Fig. 5A). Gene mutations have been widely reported to be highly accurate indicators of immunotherapy response^55^. Figure S6A showed the 20 genes with the largest mutation frequencies in HCC, including *TP53*, *TTN*, and *CTNNB1*. We then compared the alterations in mutational frequencies between the two clusters and found that *TP53* was more frequently mutated in C2, compared to C1, while the opposite was true for *CTNNB1* (Figure S6B). Notably, we observed additional mutational co-occurrence and mutually exclusive mutation events in C1 (Figure S6C-D).

We further calculated that the status of immune cell invasion in the two clusters was also very different, including B cell naive, B cell memory, T cell regulatory, NK cell resting, Monocyte, and Macrophage M0 (*P* < 0.05, Fig. 5B). Surprisingly, we found that C2 exhibited relatively high levels of ICP (*P* < 0.05, Fig. 5C), which is inconsistent with the meaning of the TIDE score and may imply that C2 belongs to a unique subtype of immune exclusion, which was characterized by a high degree of fibrosis. Previous results suggested that *FSTL3*, *PKM*, *TMEM51*, and *SERPINC1* may be hub genes that influenced differences in pathological features intrinsic to the calcium phenotype.

Considering the significant TIDE score difference between the two clusters, we calculated the correlation between the *FSTL3*, *PKM*, *TMEM51*, *SERPINC1* genes and TIDE score respectively and observed that the TIDE score was directly associated with a high *FSTL3*, *PKM*, *TMEM51* while negatively correlated with *SERPINC1* (*P* < 0.001, Fig. 5D). Based on TCGA-LIHC, there was high expression of *FSTL3* and *PKM* in the HCC tumor samples compared with the controls (*P* < 0.05, Fig. 5E). We observed at the pan-cancer level that the expression pattern of *FSTL3* and *PKM* varied greatly among different cancer types (Fig. 5F). Considering that there has been an explosive growth in research on *PKM* in cancer, we further focus on *FSTL3* and confirmed the abnormal expression of *FSTL3* in two independent validations set (*P* < 0.0001, Fig. 5G). To establish a link between FSTL3 and calcium channels, spatial transcription data in GSE203612 were obtained to characterize the spatial overlap of *FSTL3* and calcium channel-encoding genes on HCC cancer tissues. We observed that *FSTL3*, *ITPR2*, *ITPR3*, *TPCN1*, and *TPCN2* showed similar spatial distributions (other calcium channel-encoding genes are under-expressed or not expressed at all), implying potential co-expression of *FSTL3* and calcium channels (Figure S7A). We found that *FSTL3* and most calcium channel-encoding genes are significantly positively correlated at the transcriptional level. Finally, we confirmed the co-localization of FSTL3 and TPCN1, ITPR3 by IF staining in HCC tissues (Figure S7C).

Given that our estimated results suggested that *FSTL3* has potential association with worse immunotherapeutic prognosis, we gathered clinical data from 40 HCC patients for further study. The average H-SCOREs for FSTL3 content in HCC were higher than that in paracancerous tissue (Fig. 5H–I; *P* < 0.001, ANOVA). Interestingly, the *FSTL3* content was also upregulated in C2 (*P* = 8.1e−13, Fig. 5J). Further calculations showed that *FSTL3* was independent of MSI but inversely associated with TMB (r = − 0.244, *P* < 0.001, Fig. 5K–L). Notably, *FSTL3* was also observed to positively correlate with *PDCD1* levels (r = 0.377, *P* < 0.001, Fig. 5M), which indicates that patients with elevated *FSTL3* levels are more likely to benefit from immunotherapy. Finally, we collected 40 HCC samples and performed IHC staining to assign scores to FSTL3 expression levels, showing that responsive patients had lower FSTL3 expression than those non-responders (*P* = 0.023, Fig. 5N), and that patients with elevated *FSTL3* levels had a reduced response rate, compared to patients with reduced *FSTL3* levels (Fig. 5O). This part of the results strongly suggested that *FSTL3* has some diagnostic value and was closely associated with immunotherapy response.

### Identification of FSTL3 as a potent promoter of fibroblast activation

**Figure 6:**
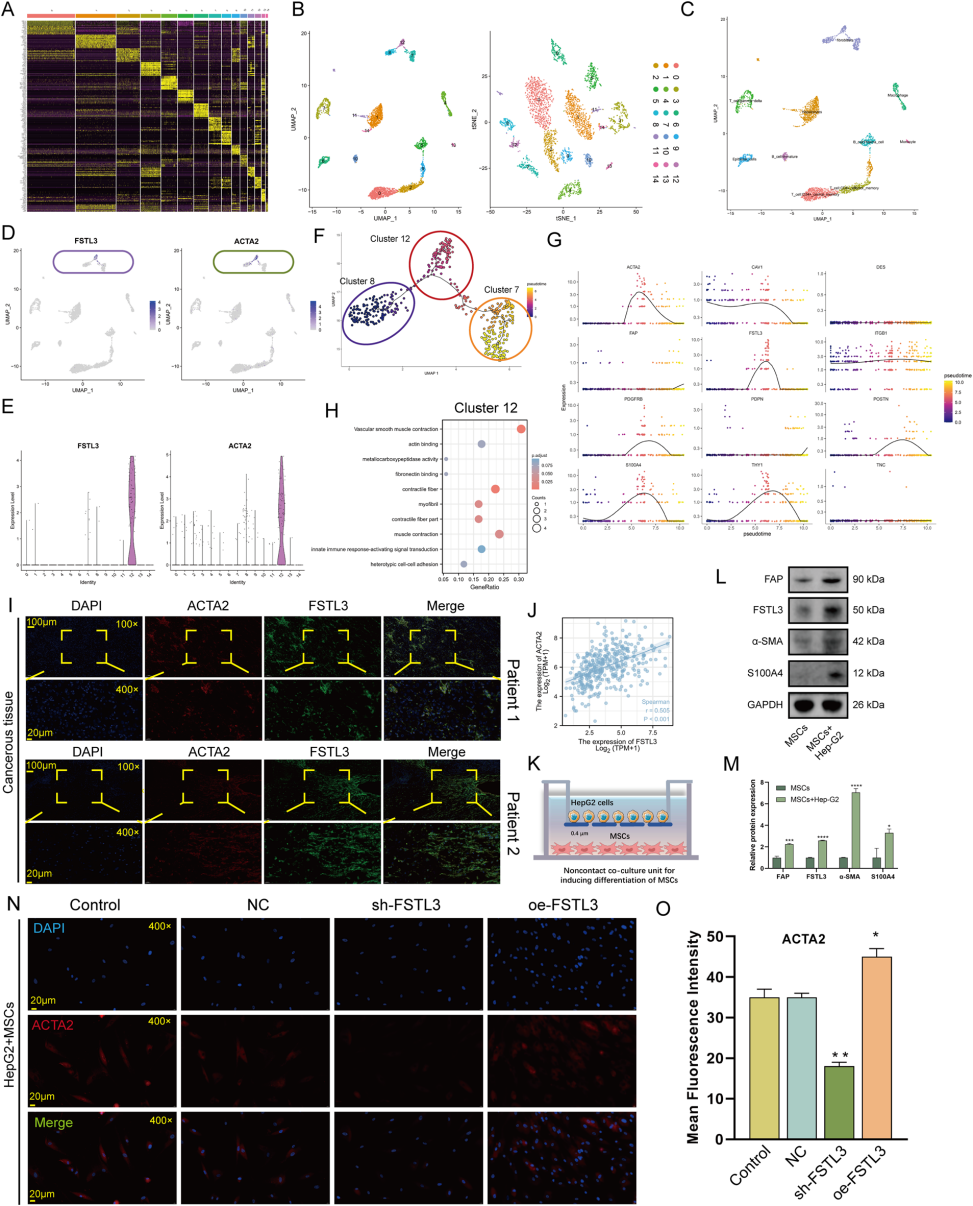
Evaluation of the relationship between *FSTL3* and response to immunotherapy. (**A**) Cell-type markers. The relative cell type-sorted gene expression across all cells. Cell-type marker genes were recognized using an unbiased method (Wilcoxon rank-sum test, FDR < 0.01, and fold change > 1.5) and the leading 15 genes are presented. (**B**) The Uniform Manifold Approximation and Projection (UMAP) and t-Distributed Stochastic Neighbor Embedding (t-SNE) plot of all high-quality cells for visualization of cell clusters. (**C**) Dot plot depicting the post manual modification condition, with individual colors indicating annotated cell types of individual clusters depending on the content of known marker genes. (**D**,**E**) Dot plots (**D**) and violin plot (**E**) illustrate the profile of FSTL3 and fibroblast activation marker gene ACTA2 expression. (**F**) Pseudotime trajectory of all fibroblasts. Circle with distinct colors indicate distinct Seurat-identified clusters and all fibroblasts were colored according to their assigned pseudotime values. (**G**) Jitter plots depicting the fibroblast activation markers and *FSTL3* profile alterations over pseudotime. (**H**) Pathway enrichment analysis of Cluster 12. (**I**) FSTL3 and ACTA2 double immunofluorescence-stained images within HCC tissue. FSTL3 and ACTA2 co-staining is presented in the enlarged images below. Scale bars, 100 and 20 mm (enlarged images). Nuclei (DAPI) in blue. (**J**) Correlation between *FSTL3* and *ACTA2* (Spearman method, TCGA-LIHC, n = 370). (**K**) Non-contact MSCs and HepG2 cell co-culture in a 1:1 ratio. (**L**,**M**) Activation markers of CAFs and expression FSTL3 in MSCs was evaluated by Western blotting (n = 3 replicates), the full uncut gels in Figure S14. (**N**,**O**) immunofluorescence of untreated CAFs and CAFs incorporated with NC, sh-FSTL3, and oe-FSTL3 constructs (magnification, × 400, scale bars = 20 μm). Immunofluorescence intensity is presented as mean ± SEM (n = 3 replicates).

New techniques in SC profiling studies will contribute to an enhanced comprehension of the microenvironmental features during HCC occurrence. To better characterize the *FSTL3* expression profile in TME, we first performed a SC level analysis based on GSE125449. The gene marker of each cluster was shown in Fig. 6A, and the SC analysis process was depicted in Fig. 6B with a UMAP and t- t-SNE that showed the distribution of individual cell clusters. We next obtained the specific cell types by unbiased annotation (Fig. 6C). The *FSTL3* expression at the SC level was illustrated in Fig. 6D, which was consistent with the fibroblast marker gene *ACTA2* (Fig. 6D,E), indicating that *FSTL3* was mainly expressed on fibroblasts. CytoTRACE analysis indicateed that cluster 7 exhibited significantly less differentiated state than other fibroblasts cell populations (Figure S8A-B, starting point of fibroblast differentiation). With pseudotime inference, we found that there were two distinct evolutionary trajectories in the trajectory process of fibroblasts, including cluster 12 and cluster 7 (Fig. 6F). We then selected *FSTL3*, 11 fibroblast activation markers (*ACTA2*, *S100A4*, *FAP*, *TNC*, *POSTN*, *DES*, *PDGFRB*, *THY1*, *PDPN*, *ITGB1*, *CAV1*), and 43 calcium channel molecules (13 members were not shown due to extremely low expression levels making them unavailable for analysis) and observed the changes in their expression levels during fibroblast differentiation by pseudotime analysis. No significant changes were observed in the expression of 30 calcium channel molecules (Figure S9). Among fibroblast activation markers, the trends of *ACTA2*, *CAV1*, *PDGFRB*, *THY1*, *S100A4* were consistent with *FSTL3*, suggesting that *FSTL3* may be critical for fibroblast activation (Fig. 6G and Figure S10). Functional enrichment analysis showed that cells in cluster 12 were enriched for fibrosis-related genes (Fig. 6H). IF staining results further validated that the *FSTL3* levels were highly consistent with *ACTA2* in cancerous and paracancerous tissue (Fig. 6I and Figure S11).

TCGA-LIHC-based calculations revealed a strong associatiBon between *FSTL3* and *ACTA2* (Fig. 6J). It is well established that local and recruited Mesenchymal stem cells (MSCs) differentiates into cancer-associated fibroblasts (CAFs) at close proximity to tumor cells. Fibroblast activation protein (FAP), fibroblast-specific protein-1 (FSP-1, S100A4), and α-smooth muscle actin (α-SMA, ACTA2) are commonly employed for activated CAFs labelling. We co-cultured MSCs with HCC cells (Fig. 6K), and the significant increase in activation markers of CAFs indicated that MSCs were successfully induced into CAFs (Fig. 6L). When MSCs were induced into CAFs, there was a significant increase in the expression of FSTL3. Based on IF staining, α-SMA contents were strongly elevated in *FSTL3-*overexpressed cells, and they were markedly diminished in the sh-*FSTL3-*treated cells (Fig. 6M–O). These results suggested that *FSTL3* is a potent promoter of fibroblast proliferation and activation in HCC.

### Identification of *FSTL3* association with M2 Macrophages

**Figure 7:**
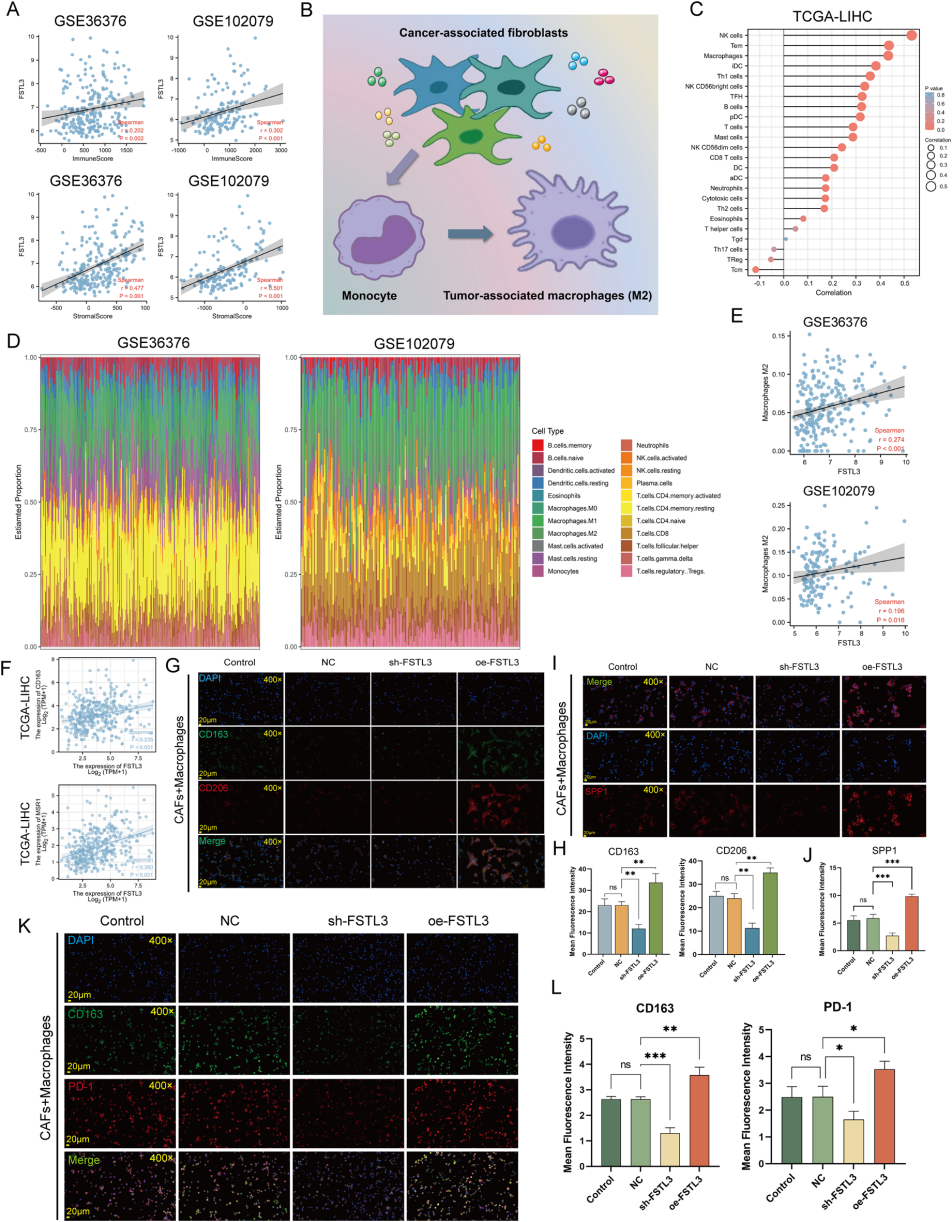
Analysis of the relationship between FSTL3 and immunosuppressive cells. (**A**) The FSTL3 transcript levels and stromal/immune score correlation in the GSE36376 (n = 433) and GSE102079 datasets (n = 257). (**B**) Schematic of tumor-associated macrophages (TAMs) and cancer-associated fibroblasts (CAFs) communication. (**C**) Spearman correlation between *FSTL3* content and 24 immune cell types using the TCGA-LIHC dataset (n = 370); positive and negative associations are marked by red and blue lollipops, respectively. (**D**) The 22 tumor-initiating cell (TICs) type profiles within HCC samples from GSE36376 (left, n = 433) and GSE102079 (right, n = 257) data. (**E**) The *FSTL3* content and M2 macrophage abundance association within the GSE36376 and GSE102079 data. (**F**) The FSTL3 content and M2 macrophage markers (CD163 and MSR1) association within the TCGA-LIHC dataset (n = 370). (**G**,**H**) Establishment of a CAFs and Macrophages Co-culture System, M2 macrophage markers CD206 (red) and CD163 (green) double-immunofluorescence staining; nuclei were DAPI-stained (blue) (Magnification, × 400, scale bars = 20 μm) (n = 3 replicates). Immunofluorescence intensity (mean ± SEM) (n = 3 replicates). Data presented as means ± SEM, **p < 0.01. (**I**,**J**) Immunofluorescence staining images of SPP1 macrophage in the Co-culture System. SPP1 (red). Scale bars, 100 and 20 mm (enlarged images). Nuclei (DAPI) in blue. Immunofluorescence intensity (mean ± SEM) (n = 3 replicates). Data presented as means ± SEM, ***p < 0.001. (**K**,**L**) Double immunofluorescence staining images of M2 macrophage in the Co-culture System. CD163 (green) and PD-1(red). Scale bars, 100 and 20 mm (enlarged images). Nuclei (DAPI) in blue. Immunofluorescence intensity (mean ± SEM) (n = 3 replicates). Data presented as means ± SEM, *p < 0.05, ***p < 0.001.

Fibroblasts are now found to be highly correlated with interstitial fibrosis of tumor, representing a poor prognosis. Using ESTIMATE analysis, we demonstrated that *FSTL3* was positively associated with immune (*P* = 0.002, r = 0.202; *P* < 0.001, R = 0.302; Fig. 7A) and stromal score (*P* < 0.001, r = 0.477; *P* < 0.001, R = 0.501; Fig. 7A) in the GSE36376 and GSE102079 datasets, respectively. In a manner of growth factors exchange, macrophages and fibroblasts do indeed form a two-cell circuit (Fig. 7B). Since we have demonstrated the role of FSTL3 in fibroblasts, we then conducted ssGSEA to profile the overall immune and stromal infiltration levels in association with *FSTL3* level, and the result exhibited an extremely strong positive correlation between *FSTL3* and macrophages (Fig. 7C). Subsequently, 22 immune cell profiles for HCC samples were generated for assessing the relationship of *FSTL3* levels with macrophage polarization (Fig. 7D) and we observed that the expression level of *FSTL3* was directly associated with M2 macrophage abundance (r = 0.274, *P* < 0.001, GSE36376; r = 0.196, *P* = 0.016, GSE102079; Fig. 7E). We also calculated the association between *FSTL3* and M2 macrophage markers according to TCGA-LIHC and noted that *FSTL3* levels were positively associated with *CD163* (r = 0.235, *P* < 0.001, Fig. 7F) and *MRC1* (r = 0.380, *P* < 0.001, Fig. 7F). To further examine the impact of *FSTL3* overexpression on M2 macrophage abundance in HCC, we developed a fibroblast–macrophage co-culture model and revealed that *FSTL3* overexpression strongly elevated the surface markers of M2 macrophage (CD206 and CD163) in THP-1 macrophages (Fig. 7G–H). We used clinical samples to conduct IF and found that CD163 and CD206 were also significantly highly expressed in the high FSTL3 expression region and was scarcely expressed with reduced FSTL3 expression (Figure S12). A recent study showed that SPP1 + macrophages and cancer-associated fibroblasts can stimulate extracellular matrix remodeling and promote Tumor Immune Barrier (TIB) formation^56^. Thus, we performed IF in CAFs and Macrophages Co-culture System. The abundance of SPP1 + macrophages changes with the expression level of FSTL3 in CAFs (Fig. 7I–J). As a group of cells that influence the effectiveness of immunotherapy, macrophages express PD-1 on their membranes and thereby mediate the immune escape of tumor cells^57^. Given that we have demonstrated a correlation between FSTL3 and PD-1 and that FSTL3 promotes the proliferation of M2-like macrophages, we further investigated whether FSTL3 is involved in the regulation of PD-1 expression. We found that FSTL3 was able to upregulate PD-1 expression on M2- like macrophages (Fig. 7K,L). In addition, in CAFs and HepG2 Co-culture System, the expression of PD-L1 can be altered by FSTL3 in CAFs (Figure S13). Together, these results suggest that FSTL3 regulates immunosuppression in TME in at least a fibroblast-macrophage axis-dependent manner.

## Discussion

HCC remains a global health challenge. Due to its insidious onset and mild symptoms in the early stages, more than half of patients are diagnosed with tumors that are too large or have spread to distant organs to be treated surgically^58^. In these cases, hepatic artery chemoembolization, regional radiotherapy and molecular targeted therapy are not effective^59^. The combination of targeted and immunotherapy has ushered in a new era of liver cancer treatment. However, it still faces the problems of uncertain efficacy, low objective remission rate, many side effects, and patients may develop drug resistance even after benefit^60^. Therefore, to guide the selection of HCC treatment regimens based on molecular typing, so that more HCC patients can benefit from it, is the challenge to be solved and the direction of the future development of precision therapy for HCC.

The ion Ca(2 +) acts as a widespread second messenger in the body and mediates a variety of cellular functions throughout the cell cycle^61^. Numerous studies have confirmed that imbalance of calcium channel homeostasis underlies the pathology of many diseases, including tumors, particularly in the context of the important mechanism of Store-operated Ca(2 +) entry (SOCE)^62–64^. Ca^2+^-mediated signaling pathways play an important role in cellular phenotypic transformation, such as the transformation of normal cells to cancer cells, tumor formation and growth, invasion, angiogenesis, and metastasis^65–68^.

Considering that previous studies were mostly limited to the promotion or inhibition of tumor development by individual calcium channels and the heterogeneity of calcium channel expression in individuals with HCC, we clustered the TCGA-LIHC cases based on the expression profiles of all 43 known calcium channel-encoding genes and confirmed that calcium channels could effectively stratify patients with HCC. To ensure the reliability of this subtype definition as well as its reproducibility, we subsequently replicated it in the GEO dataset. We observed marked alterations in clinical features and statuses of immune cell infiltration between clusters. The biological behavior of both innate and adaptive immune cells have been proved to be tightly regulated by a cascade of signals provided by different ion channel networks in previous studies^69^. Our study demonstrated a strong link between overall calcium channel levels and TME.

Recent studies have shown that Ca(2 +) oscillations are critical for regulating gene expression in fibroblasts and it is now known that, on the one hand, pulsatile released Ca(2 +) promotes a voltage-dependent Ca(2 +) influx pathways by activating membrane conductance and, on the other hand, calcium signaling acts upstream of mechanoenzyme, thereby mediating cellular rearrangements^70,71^. The above factors ultimately coordinating a large number of cellular events, particularly the synthesis/secretion of ECM proteins and activation of fibroblasts^72^. As the major reservoir of intracellular Ca2 + , the endoplasmic reticulum (ER) releases Ca2 + into the cytoplasm and mitochondria under the control of IP3Rs (encoded by ITPR3)^73^. Ca2 + release promotes the formation of mitochondrial ATP, which regulates cellular energy metabolism^74^. Calcium channel-stimulated Ca2 + release has a significant effect on fibroblast behavior, not only promoting discrete reorganization of actin filaments and thus altering cell morphology, but also regulating secretion of fibroblast matrix metalloproteinases^75^. These facts influence the secretion of collagen by fibroblasts and remodel the structure of the extracellular matrix. Based on these perceptions, and to clarify the underlying causes of the different clinical outcomes between the two subtypes, we revealed an interesting phenomenon through histopathological sections: patients in C1 had a lower matrix content in the cancer tissue compared to C2, while the C2 subtype was highly fibrotic and formed dense collagen. Considering the unique pathological features of the C2 subtype, we further analyzed the biological functional differences between the two subtypes and found that the differential genes were mainly enriched in ECM and collagen formation-related pathways. To precisely identify key genes in differential genes, we used the MEGENA algorithm to obtain core gene modules and identified *FSTL3*, *TMEM51*, *PKM*, and *SERPINC1* as potential pivotal genes based on degree ranking and Kaplan Meier analysis, where the levels of *FSTL3*, *TMEM51* and *PKM* were positively correlated with TIDE, while the *SERPINC1* expression was inversely associated with TIDE. We further observed overexpression of FSTL3 and PKM in HCC tissues, suggesting their possible involvement in HCC development. The significance of PKM, a key enzyme in the final rate-limiting step of glycolysis, in tumor cell metabolism has now been extensively studied^76,77^, whereas the potential function of FSTL3, an oncogene that has recently piqued the interest of researchers, in HCC has not been elucidated^78,79^. We further validated the aberrant expression of FSTL3 in HCC tissues with independent microarray datasets as well as additional clinical specimens and confirmed that HCC patients with elevated FSTL3 levels are less responsive to immunotherapy compared to patients with reduced *FSTL3* levels. The work of Chao Yang et al. showed that *FSTL3* can serve as a bioindicator of ECM remodeling in colorectal cancer and correlates with chemoresistance, and in particular they reported an extremely strong association between *FSTL3* and CAFs^80^.

Emerging evidences indicate that, tumor matrix components, and in particular CAFs, are essential modulators of immunotherapeutic resistance^81^. It is a basic fact that the effectiveness of immunotherapy is mainly influenced by T-cell dysfunction, which refers mainly to the abundance of T cell invasion and the quality of T cells, and by T cell exclusion, which depends mainly on the abundance of immunosuppressive cells namely, CAFs and M2 macrophages that restrict T cell infiltration in TME^81–84^. Meanwhile, due to the unique phenomenon of tumor fibrosis dominated by CAFs, most solid tumors exhibit three main immune phenotypes due to the heterogeneity of their intrinsic components, including immune inflamed, immune desert, and immune excluded^85^. FSTL3 specifically binds TGF-β superfamily members, inhibits Smad family protein-mediated intracellular signaling, thus affecting target gene expression^86^. Ankur Chakravarthy et al. demonstrated that the activation of TGF-β signaling is a guiding factor which links CAFs to immune evasion and ECM transcriptional programme dysregulation^87^. As no previous studies have reported the localization and function of *FSTL3* in HCC, we subsequently performed a SC analysis and found that *FSTL3* was ubiquitously present on fibroblasts with high *ACTA2* expression, and the IF results further confirmed the co-localization of FSTL3 with ACTA2. Subsequently, the pseudotime analysis showed a consistent expression trend of *FSTL3* and fibroblast activation associated genes. It is also noteworthy that *FSTL3* highly enriched cell cluster (cluster 12) was characterized by a significant fibrosis-related phenotype. These results implied a potential function of *FSTL3* in fibroblast activation. To verify this inference, we constructed a cell line of CAFs stably transfected with *FSTL3* and confirmed that *FSTL3* can significantly promoted the activation of CAFs. In TME, CAFs secrete large amounts of ECM molecules, such as collagen and fibronectin, leading to intense tumor tissue remodelling. It is therefore now generally accepted that the intratumoral fibrotic response originates from the activation and proliferation of CAFs. Corresponding fibrillar conformational changes enhance tumor-stromal interactions, triggering a malignant phenotype of cancer cells by promoting cell dedifferentiation and cancer stem cell division. From this point of view, the over-activation of FSTL3 in C2 may be a contributing factor to collagen matrix accumulation.

The complex signaling crosstalk that exists between fibroblasts and macrophages makes their malignant behavior highly consistent in many ways, especially in terms of weakening the local immune response of the body^88,89^. The ssGSEA and CIBERSORT revealed that *FSTL3* was highly associated with macrophage abundance, especially M2 macrophage, and these evidences indicated that *FSTL3* may exert immunosuppressive effects through T-cell exclusion-related pathways. We finally confirmed by IF that *FSTL3* expressed by CAFs could stimulate the proliferation of M2 macrophages, and that the expression of M2 macrophage marker proteins (CD163, CD206) increased in parallel when the local area had a relatively high level of *FSTL3* expression. M2 macrophages, a recognized anti-inflammatory component, are able to synergize with the over-abundant collagenous tissue secreted by fibroblasts to jointly block immunotherapeutic agents from reaching the tumor component^90–92^. In addition, macrophages, one of the key cells that express PD-1 within tumors, are able to attenuate T-cell responses and are currently considered as biomarkers for determining therapeutic response^93,94^. Our results confirm that FSTL3 in the stroma component may play an important role in immune checkpoint inhibition.

The present study also has some limitations. Firstly, the basic findings are based on computer simulation methods, although validated in their own samples, however, due to a relatively small sample population, there is a need to expand the study to obtain more adequate evidence. Second, since immunofluorescence staining is difficult to assess samples quantitatively, a more rigorous approach would be appropriate accordingly. Finally, the specific functional localization of *FSTL3* in tumors remains ambiguous, and there is an urgent need to expand the scope and methods of studies targeting it.

## Conclusion

Our findings suggested that calcium channel molecules are closely associated with hyperfibrosis in TME of HCC patients, while we identified the fibroblast-associated gene *FSTL3* in a highly mesenchymal population. *FSTL3* is associated with CD163/CD206 M2 macrophages, further contributing to anti-inflammatory effects and leading to immunotherapy resistance. Further studies of the fibroblast/FSTL3/M2 macrophage axis will help to provide potential new targets for HCC immunotherapy.

### Supplementary Information

Supplementary Information.

## Data Availability

The data and materials in the current study are available from the corresponding author: zxvery@126.com.

## Abbreviations

HCC: Hepatocellular carcinoma
TCGA: The Cancer Genome Atlas
MEGENA: Multiscale Embedded Gene Co-expression Network Analysis
TIDE: Tumor Immune Dysfunction and Exclusion
IHC: Immunohistochemistry
IF: Immunofluorescence
GEO: Gene Expression Omnibus
FSTL3: Follistatin-like 3
TME: Tumor microenvironment
mAb: Monoclonal antibodies
FPKM: Fragments per kilobase million
UCSC: University of California, Santa Cruz
LIHC: Liver hepatocellular carcinoma
GTEx: Genotype-Tissue Expression
HGNC: Human Gene Nomenclature Committee
DEGs: Differentially expressed genes
PFN: Planar filtered network
MCA: Multiscale clustering analysis
TMB: Tumor mutational burden
MSI: Microsatellite instability
GO: Gene ontology
KEGG: Kyoto encyclopedia of genes and genomes
ICB: Immune checkpoint blockade
scRNA-seq: Single-cell RNA sequencing
UMAP: Uniform Manifold Approximation and Projection
t-SNE: t-distributed stochastic neighbor embedding
UMI: Unique molecular identifier
EMT: Epithelial-mesenchymal transition
AR: Androgen receptor
MAPK: Mitogen-activated protein kinase
RTK: Receptor tyrosine kinases
CNV: Copy-number variation
OS: Overall survival
DSS: Disease-specific survival
ECM: Extracellular matrix
MSCs: Mesenchymal stem cells
CAFs: Cancer-associated fibroblasts
FAP: Fibroblast activation protein
FSP-1: Fibroblast-specific protein-1
α-SMA: α-smooth muscle actin
SOCE: Store-operated Ca(2+) entry
