# Supplementary material for: Stroma-associated FSTL3 is a factor of calcium channel-derived tumor fibrosis
Source: Sci Rep. 2023 Dec 3;13:21317. doi: 10.1038/s41598-023-48574-8 (PMC10694158; doi:10.1038/s41598-023-48574-8)
Supplement: Supplementary file 1 — Supplementary Information. [file 41598_2023_48574_MOESM1_ESM.docx]

**Supplementary materials**

**Antibodies, reagents**

| Antibodies and Reagents | Manufacturer, Country, Batch number | Concentration |
| --- | --- | --- |
| Lentiviral vectors of FSTL3 | GeneChem, China, #136427D | - |
| Polybrene | GeneChem, China. #139146B | 2 μg/mL |
| Puromycin | Beyotime Biotechnology, China, #041321210517 | 1.5 μg/mL |
| Radioimmunoprecipitation assay buffer (RIPA buffer) | Beyotime Biotechnology, China，P0013B | - |
| Sodium dodecyl-sulfate polyacrylamide gel electrophoresis (SDS-PAGE) | EpiZyme, China, #LK209 | - |
| Tricolor Prestained Protein Marker | EpiZyme, China, #WJ103 | - |
| Anti-FSTL3 Antibody | Invitrogen, USA. #SI2436032 | WB 1:1000  IF:500  IHC:500 |
| anti-CD163 polyclonal antibodies | Abcam, Cambridge, UK, #ab87099 | IF 1:1000 |
| CD206 Polyclonal Antibody | Invitrogen, USA, #PA5-46994 | IF 1:500 |
| Alpha-Smooth Muscle Actin Polyclonal Antibody | Invitrogen, USA, # PA5-117765 | WB 1:1000  IF 1:500 |
| Anti-GAPDH Mouse polyclonal Antibody | Abbrab, China, #AB300016 | WB 1:3000 |
| Anti-FAP Antibody | Invitrogen, USA, #30E12A05 | WB 1:1000 |
| Anti-SPP1 Polyclonal Antibody | Solarbio, China, # K006808P | IF 1:100 |
| PD-L1/CD274 (C-terminal) Polyclonal antibody | Proteintech, China, # 28076-1-AP | IF 1:200 |
| ITPR2 Polyclonal antibody | Proteintech, China, # 20730-1-AP | IF 1:200 |
| ITPR3 Polyclonal Antibody | Invitrogen, USA, # PA5-88758 | IF 1:200 |
| Anti-S100A4 Antibody | Invitrogen, USA, #MAB02754 | WB 1:1000 |
| phorbol 12-myristate 13-acetate (PMA) | Sigma-Aldrich, USA, #SLBX8899 | 10 ng/mL |
| Goat anti-Rabbit IgG (H&L) conjugated with Alexa Fluor® 594 | Abcam, Cambridge, MA, USA, # ab150084 | 1:2500 |
| Goat anti-Mouse IgG (H&L) conjugated with Alexa Fluor® 488 | Abcam, Cambridge, MA, USA, # ab150113 | 1:2500 |
| Nuclei were stained using 4',6-diamidino-2-phenylindole (DAPI) | Beyotime Biotechnology, China, # C1005 | - |
| Dulbecco’s modified Eagle’s medium (DMEM) | Gibco, USA, # 8121032 |  |
| Roswell Park Memorial Institute 1640 (RPMI-1640) | Gibco, USA, # 8121248 |  |
| Fetal bovine serum (FBS) | Gibco, USA, #42F1376K | - |

# Supplementary Method

- 1. **Inclusion/exclusion criteria for participants**

In this retrospective analysis, the patients with HCC archival materials were obtained from the Department of Pathology of Jiangsu Province Hospital of Chinese Medicine, Affiliated Hospital of Nanjing University of Chinese Medicine. None of the patients underwent chemotherapy, radiotherapy and adjuvant treatment prior to surgery. All patients were followed up by consulting their case documents and by telephone. We included unselected consecutive patients with histologically confirmed HCC that had been treated with Immune checkpoint inhibitors in combination with sorafenib at Jiangsu Province Hospital of Chinese Medicine Affiliated Hospital of Nanjing University of Chinese Medicine between 2019-05-01 and 2022-05-01. A total of 40 cases met the inclusion criteria for this retrospective study. All patients were diagnosed by clinical examination, ultrasonography (US), magnetic resonance imaging (MRI), PET-CT/CT, angiography and hematology. The resected samples of all patients were confirmed by pathological examination. Those patients were treated with immune checkpoint inhibitors in combination with sorafenib until disease progression or unacceptable toxicity developed. We use anti-PD-1: Nivolumab (3 mg/kg every 2 weeks) or Pembrolizumab (2 mg/kg every 3 weeks); Sorafenib treatment (400 mg twice daily). Radiologic reassessment by PET-CT or CT scan was performed every 12 weeks, or as clinically indicated. Tumor response was assessed according to the Response Evaluation Criteria in Solid Tumors (RECIST), version 1.1[1].Patients were excluded if they had: (1) patients whose pathological diagnosis was not HCC, such as cholangiocarcinoma (CCC); (2) patients who died during the treatment period; (3) patients with incomplete data or lost contact during the follow-up period; (4) infectious disease, immune system disease, blood system disease; (5) patients underwent arterial chemoembolization or Radiofrequency ablation; (6) HIV positive patients.

## Immunohistochemical staining and Tissue Section Immunofluorescence staining

Immunohistochemical and Immunofluorescence staining followed the method described in previous study[2, 3]. The antibodies used are listed in the Supplementary Table 1. The concentrations used were based on those used in previous research or on the manufacturers’ recommendations. After blocking tissue sections with protein blocking solution, slides were incubated with primary antibody. IHC results (intensity and extent of staining) were independently scored by two observers. The images were captured using a NIKON Eclipse Ni-E microscope (NIKON, Japan). Staining intensity was graded as follows: 0, negative staining; 1, weak staining; 2, moderate staining; and 3, strong staining. The extent of staining was scored based on the proportion of positively stained cells per specimen, as follows: 0, no positively stained cells; 1 <10% positively stained cells; 2, 10–50% positively stained cells; and 3, >50% positively stained cells. The histochemistry score (H-SCORE), which represents the proportion of positively stained cells and the intensity of expression, was calculated as follows: H-SCORE= ∑(PI × I) = (percentage of cells with weak intensity × 1) + (percentage of cells with moderate intensity × 2) + (percentage of cells with strong intensity × 3). In the formula, PI represents the percentage of positive cells to the total number of cells in a particular field and I represent the intensity of staining. The H-SCORE ranges from 0 to 300, with a higher score representing stronger positive staining[3]. Immunofluorescence staining was performed on the tissue sections or cell climbing pieces. After blocking in 5% bovine serum albumin in phosphate-buffered saline, 0.1% Tween followed by an overnight incubation (4 °C) with the primary antibody. This procedure was followed by an incubation with the secondary antibodies. Nuclei were stained using 4',6-diamidino-2-phenylindole (DAPI) for 3 min, and the cells were incubated in the dark for 3 min. The slides were washed with phosphate buffered saline (PBS) four times, for 5 min each. Then, the slides were sealed with sealing solution containing a fluorescence quencher and observed and imaged with an inverted fluorescence microscope (Olympus CKX-41, Japan).

## Research reagents and antibodies

We have provided a complete list of reagents and antibodies in Supplementary material. All concentrations were selected based on previous studies or manufacturer's instructions.

## Cell culture

Human bone marrow Mesenchymal stem cells (MSCs) (Cat# CP-H166) and human HepG2 liver tumor model Human HepG2 cell line (Cat# CL-0103) was purchased from Procell Life Science &Technology (Wuhan, China), Human-derived monocytes (THP-1 cell line) (Cat# BNCC358410) were purchased from Beina Chuanglian Biotechnology (Beijing, China), MSCs and HepG2 cells were cultured in Dulbecco’s modified Eagle’s medium (DMEM) with 10% Fetal bovine serum (FBS). THP-1 cells were cultured in Roswell Park Memorial Institute 1640 (RPMI-1640) with 10% FBS. All cells were incubated at 37°C in 5% CO2.

## Establishment of a MSCs and HepG2 co-culture unit

The MSCs is a heterogeneous population with high differentiation ability. Local and recruited MSCs are known to convert into Cancer associated fibroblasts (CAFs) in close proximity to tumor cells. A non-contact co-culture unit of MSCs and HepG2 cells was established using a co-culture transwell system. Indirect cocultures of MSCs and HepG2 were assembled using transwell membranes (six-well plates, 0.4-μm pore size) (upper chamber: HepG2 cells, 1 × 105; lower chamber: MSCs, 1 × 105 cells). The culture medium was changed every 48 h. After 5 days of non-contact co-culture, the culture in the lower chamber was terminated and cells were harvested for other experiments. MSCs cultured without HepG2 cells were used as the control. We evaluated activation markers of CAFs such as fibroblast activated protein (FAP)、α-smooth muscle actin (α-SMA, ACTA2)、fibroblast-specific protein-1 (FSP-1) (S100A4) by western blot.

## Lentiviral Vector Construction and Transfection

We used lentiviral vectors for overexpressing and knocking down FSTL3. Viruses were designed, synthesized, and produced by GeneChem Corporation. Transfection was performed according to the supplier’s protocol.

## Construction of FSTL3 lentiviral vectors

**Easy-siRNA design:**

| **NO.** | **Accession** | **Target Seq** | **CDS** | **GC%** |
| --- | --- | --- | --- | --- |
| FSTL3-RNAi(77799-21) | NM_005860 | cgGCAACAACAACGTCACCTA | 33..824 | 47.37% |
| FSTL3-RNAi(77800-1) | NM_005860 | GATCAACCTCCTCGGCTTCTT | 33..824 | 52.38% |
| FSTL3-RNAi(77801-1) | NM_005860 | GAGCTTTGCGGCAACAACAAC | 33..824 | 52.38% |
| Description | Homo sapiens follistatin like 3 (FSTL3), mRNA | | |  |

**FSTL3-RNAi (77800-1) was chosen because it had the highest silencing efficiency**

**lentivirus vector name：** GV248

**Negative Control insert sequence:** TTCTCCGAACGTGTCACGT

**The lentivirus vector brochure can be downloaded via the following website:** http://www.genechem.com.cn/service/index.php?ac=gene&at=vector_search&keyword=GV248

**lentivirus overexpression vector name**：GV492

**The lentivirus vector brochure can be downloaded via the following website:**

http://www.genechem.com.cn/service/index.php?ac=gene&at=vector_search&keyword=GV492

**For overexpression lentiviruses, the complete nucleotide sequences of FSTL3 were constructed [5’-3’]**

[ATGCGTCCCGGGGCGCCAGGGCCACTCTGGCCTCTGCCCTGGGGGGCCCTGGCTTGGGCCGTGGGCTTCGTGAGCTCCATGGGCTCGGGGAACCCCGCGCCCGGTGGTGTTTGCTGGCTCCAGCAGGGCCAGGAGGCCACCTGCAGCCTGGTGCTCCAGACTGATGTCACCCGGGCCGAGTGCTGTGCCTCCGGCAACATTGACACCGCCTGGTCCAACCTCACCCACCCGGGGAACAAGATCAACCTCCTCGGCTTCTTGGGCCTTGTCCACTGCCTTCCCTGCAAAGATTCGTGCGACGGCGTGGAGTGCGGCCCGGGCAAGGCGTGCCGCATGCTGGGGGGCCGCCCGCGCTGCGAGTGCGCGCCCGACTGCTCGGGGCTCCCGGCGCGGCTGCAGGTCTGCGGCTCAGACGGCGCCACCTACCGCGACGAGTGCGAGCTGCGCGCCGCGCGCTGCCGCGGCCACCCGGACCTGAGCGTCATGTACCGGGGCCGCTGCCGCAAGTCCTGTGAGCACGTGGTGTGCCCGCGGCCACAGTCGTGCGTCGTGGACCAGACGGGCAGCGCCCACTGCGTGGTGTGTCGAGCGGCGCCCTGCCCTGTGCCCTCCAGCCCCGGCCAGGAGCTTTGCGGCAACAACAACGTCACCTACATCTCCTCGTGCCACATGCGCCAGGCCACCTGCTTCCTGGGCCGCTCCATCGGCGTGCGCCACGCGGGCAGCTGCGCAGGCACCCCTGAGGAGCCGCCAGGTGGTGAGTCTGCAGAAGAGGAAGAGAACTTCGTG]

## Lentiviral Vector Transfection

The target sequences for the lentivirus are summarized in Supplementary material. We used this sequence to construct a lentiviral vector expressing FSTL3 shRNA (named sh-FSTL3) and employed a non-targeting sequence lentiviral vector as the control (named NC). For overexpression, a lentivirus with the complete FSTL3 nucleotide sequence was constructed (named oe-FSTL3). CAFs were transduced with the recombinant lentivirus using 2 μg/mL polybrene for 24 h. Subsequently, we assessed FSTL3 overexpression and knockdown as well as transduction efficiency using western blots.


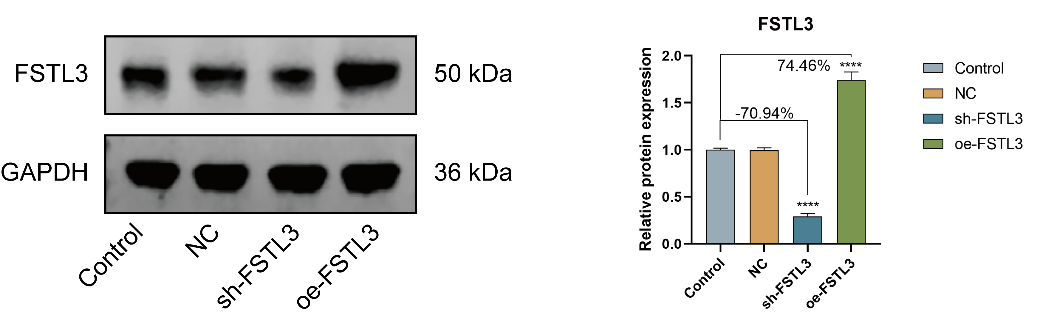


## Western blotting

Cells were lysed in RIPA buffer and protein concentrations measured by the Bradford assay. Samples of 20 µg each were separated on 10% or 8% SDS-PAGE. Proteins were transferred to polyvinylidene fluoride (PVDF) membranes and blocked with 5% bovine serum albumin. The blots were then probed with the relevant primary antibodies at 4˚C overnight. After washing three times in Tris-buffered saline containing 0.05% Tween-20, the blots were incubated with the corresponding secondary antibodies and an Electrochemiluminescence (ECL) detection kit used to measure densities. The GAPDH and β-actin protein was used as a reference.

## Puromycin screening of the stably transfected cell lines

The cell density in the 24-well plates was 5 × 10^4^ cells/well.The screening medium, i.e., fresh medium containing different concentrations of puromycin (0–10 μg/mL), was prepared. The cells were incubated overnight in a screening medium.The old screening medium was replaced with a fresh screening medium every ~2–3 days. The surviving cells were observed daily, as the optimal duration of puromycin action generally ranges from 1 to 4 days.The minimum concentration of puromycin used was the lowest concentration that kills all cells within 1–4 days from the start of the screening.After screening, a concentration of 1.5 μg/mL was chosen.

## Concentration screening with polybrene

Polybrene is a positively charged small molecule binding to the anions on the cell surface, improving the efficiency of the lentiviral transfection in the cells. The addition of polybrene can improve the efficacy of the transfection by 2- to 10-fold. Since different cells have different sensitivities to polybrene, a range of 1–10 μg/mL was used for screening at the beginning of the experiment. Finally, we settled on a working concentration of 2 μg/mL.

## Macrophages were induced from THP-1 cells.

The THP-1 cell line is a human leukemia cell-derived monocyte line that is capable of acquiring phenotypic and functional characteristics similar to those of primary macrophages upon PMA stimulation[4, 5]. It is currently the most widely used in vitro model of human-derived macrophages. It has been proven that 5 ng/mL PMA treatment can cause THP-1 monocytes to differentiate into macrophages. In addition, many researchers believe that the higher the PMA concentration, the easier it is for differentiated macrophages to transform into the M1 subtype[6]. Therefore, a low-concentration induction protocol was chosen for this study. Due to various inter-laboratory variations, initial induction with 5 ng/mL PMA for 48 h resulted in only about 60% of the THP-1 cells adhering to the wall. Subsequently, after many adjustments, 10 ng/mL for 48 h was selected as the induction model, as 95% of THP-1 cells differentiated and adhered to the wall under this treatment condition.THP-1 cells in logarithmic growth phase were centrifuged and resuspended in RPMI-1640 medium; after adding 10 ng/mL PMA to induce 48 hour, THP-1 transformed from suspension growth to wall growth, from round to irregular shape, further increased in size, cell pulp was loosened, cell nucleus enlarged obviously, a large number of obvious organelles were visible, and a small amount of protrusion around the cytosol was visible.


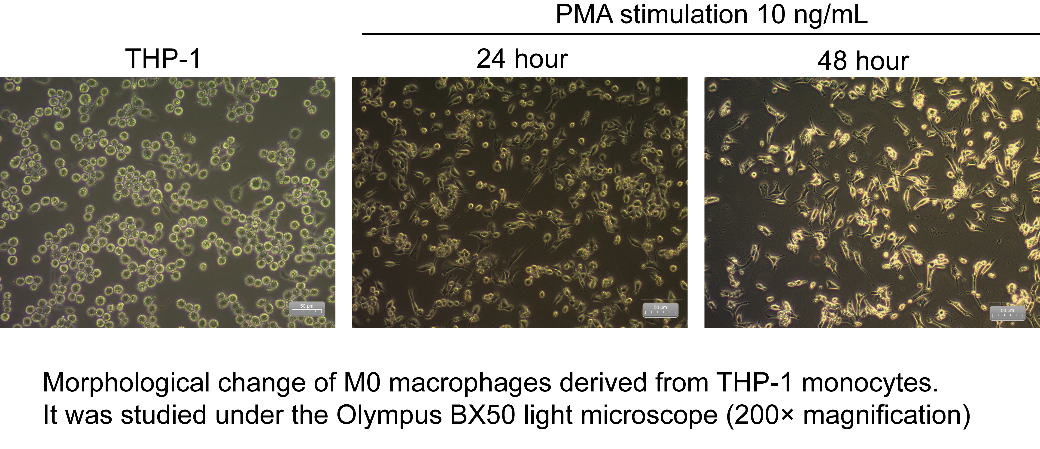


## Establishment of a CAFs and Macrophages Co-culture System

THP-1 cells (1 × 105 cells/mL) were treated with phorbol 12-myristate 13-acetate (PMA) (10 ng/mL) for 48 h to allow the induction of macrophage differentiation. PMA-containing medium was replaced with serum-free medium, and the cells were cultured for 24 hours. Two days before the co-culture experiment, CAFs (1 × 105 cells) from the control, knock-down (sh-FSTL3), overexpression (oe-FSTL3), and negative control (NC) groups were seeded onto 0.4-μM transwell inserts. For co-culture, the culture medium in the inserts with CAFs was removed and transferred to the top of the pates with differentiated macrophage cells. After 48 h of further co-culturing, cells were obtained, and immunofluorescence staining was performed. The co-culture system has been illustrated with a schematic was presented below.


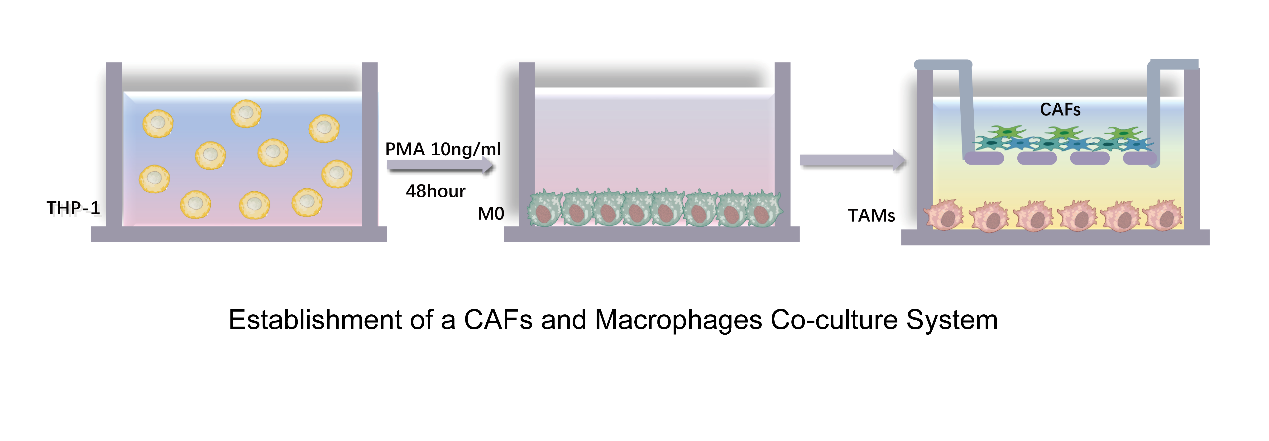


## Statistical analysis

Data are expressed as means ± SEM. Comparisons between two groups and multiple groups were assessed by t-tests and one-way ANOVA, respectively. All data were analyzed with SPSS 29.0 (IBM Corp., Armonk, NY, USA) and illustrated using GraphPad Prism 8.0 (GraphPad Software, Inc., USA). All the experiments were carried out at least thrice. ***P < 0.001, **P < 0.01 and *P < 0.05 were defined to be statistically significant.

1. Schwartz, L.H., et al., *RECIST 1.1-Update and clarification: From the RECIST committee.* Eur J Cancer, 2016. **62**: p. 132-7.

2. Im, K., et al., *An Introduction to Performing Immunofluorescence Staining.* Methods Mol Biol, 2019. **1897**: p. 299-311.

3. Magaki, S., et al., *An Introduction to the Performance of Immunohistochemistry.* Methods Mol Biol, 2019. **1897**: p. 289-298.

4. Tsuchiya, S., et al., *Induction of maturation in cultured human monocytic leukemia cells by a phorbol diester.* Cancer Res, 1982. **42**(4): p. 1530-6.

5. Genin, M., et al., *M1 and M2 macrophages derived from THP-1 cells differentially modulate the response of cancer cells to etoposide.* BMC Cancer, 2015. **15**: p. 577.

6. Chanput, W., et al., *Characterization of polarized THP-1 macrophages and polarizing ability of LPS and food compounds.* Food Funct, 2013. **4**(2): p. 266-76.

# Supplementary Figure


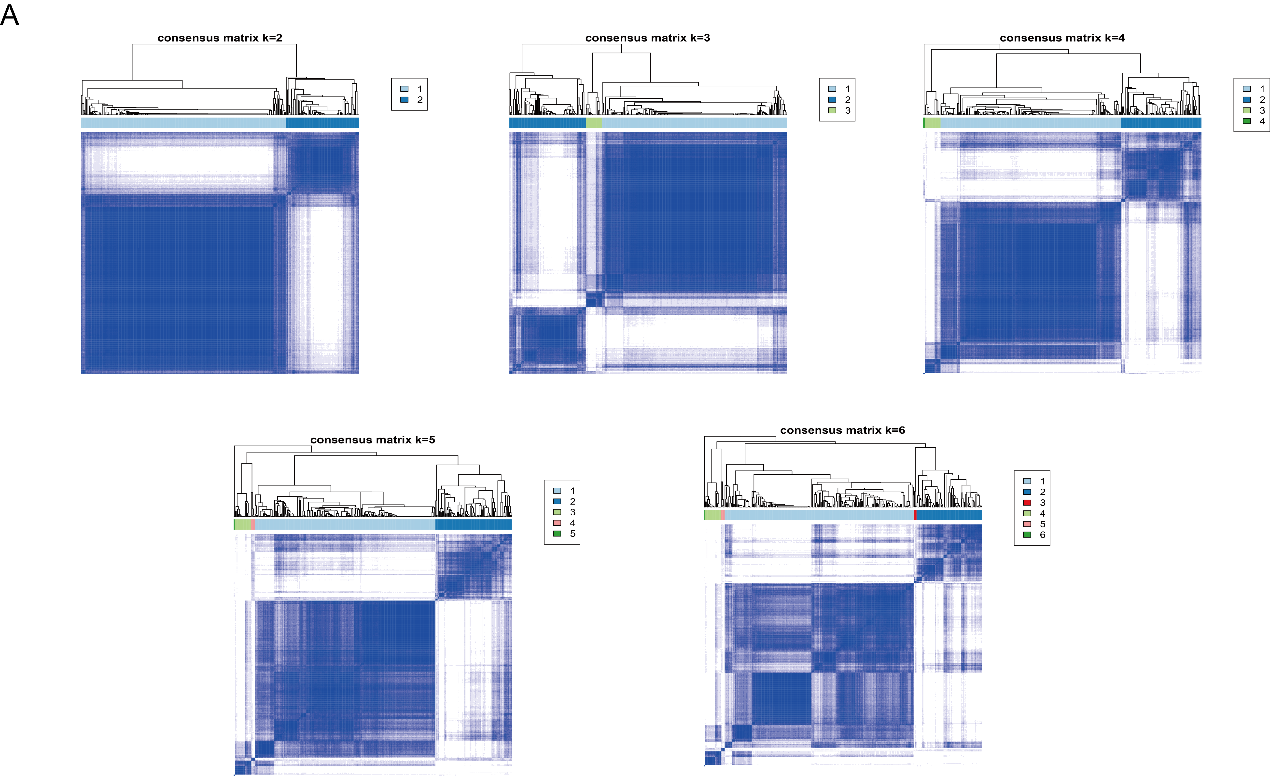


**Figure S1.**

The consensus score matrix of all samples when k = 2-6.


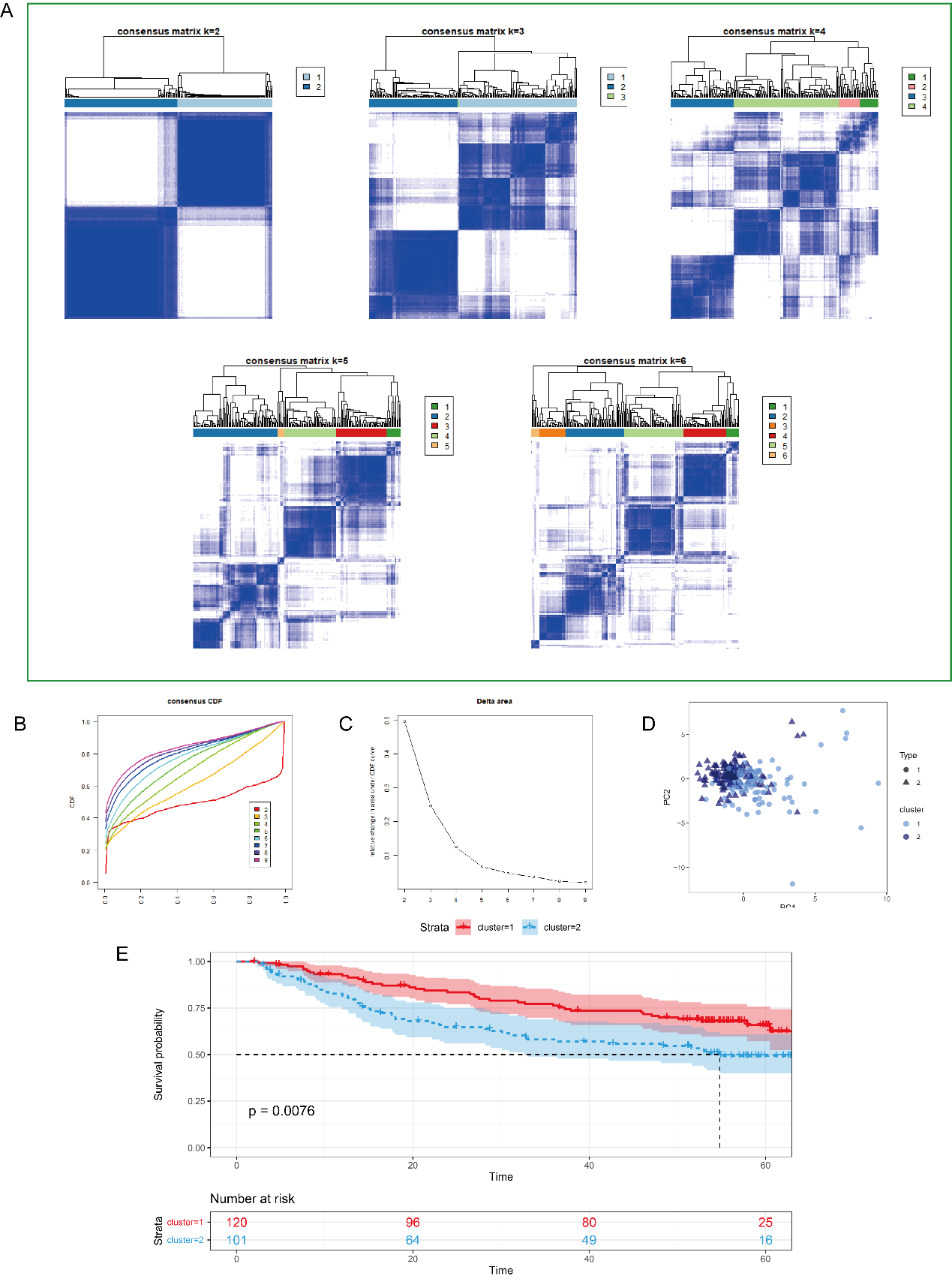


**Figure S2.**

(A)The consensus score matrix of all samples when k = 2-6. (B)The cumulative distribution function (CDF) curves in consensus cluster analysis. CDF curves of consensus scores by different subtype numbers (k = 2, 3, 4, 5, and 6) were displayed. (C) Relative change in area under the CDF curve for k = 2–6. (D) The PCA distribution of GSE14520 by expression profile of calcium channel molecules. Each point represents a single sample; different colors represent the C1 and C2 subtypes respectively. (E) Survival analysis (OS) based on two subtypes.


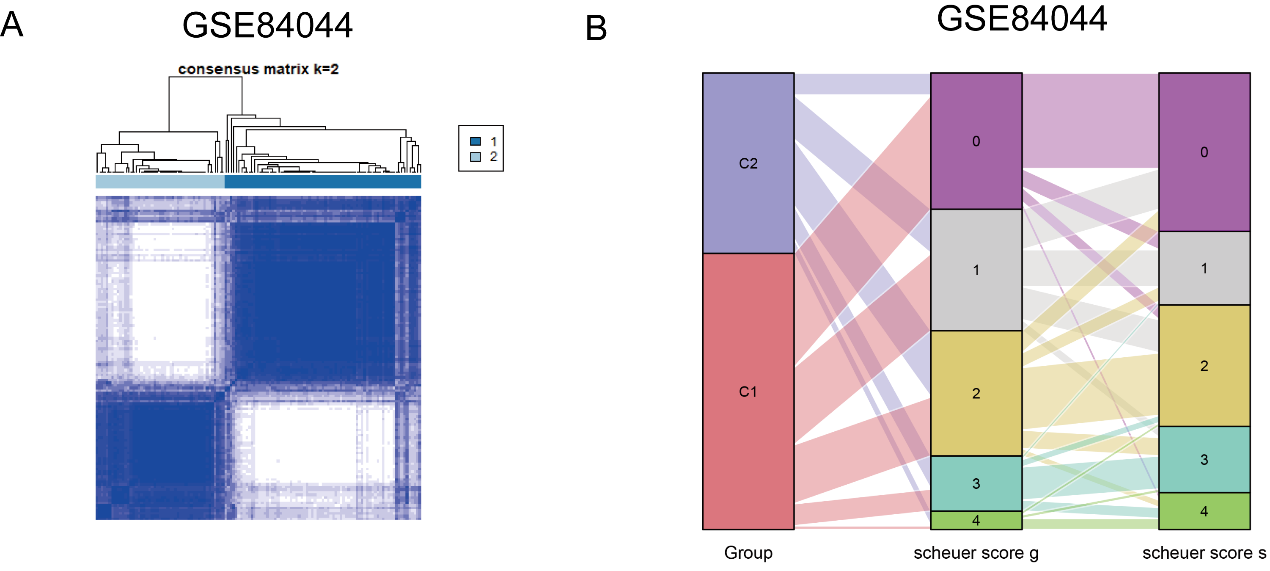


## Figure S3.

(A) The consensus score matrix of all samples when k = 2 based on GSE840044. (B) The Sankey diagram fully demonstrated the association between Scheuer staging fibrosis score, Scheuer grading inflammation score and subtypes attributes.


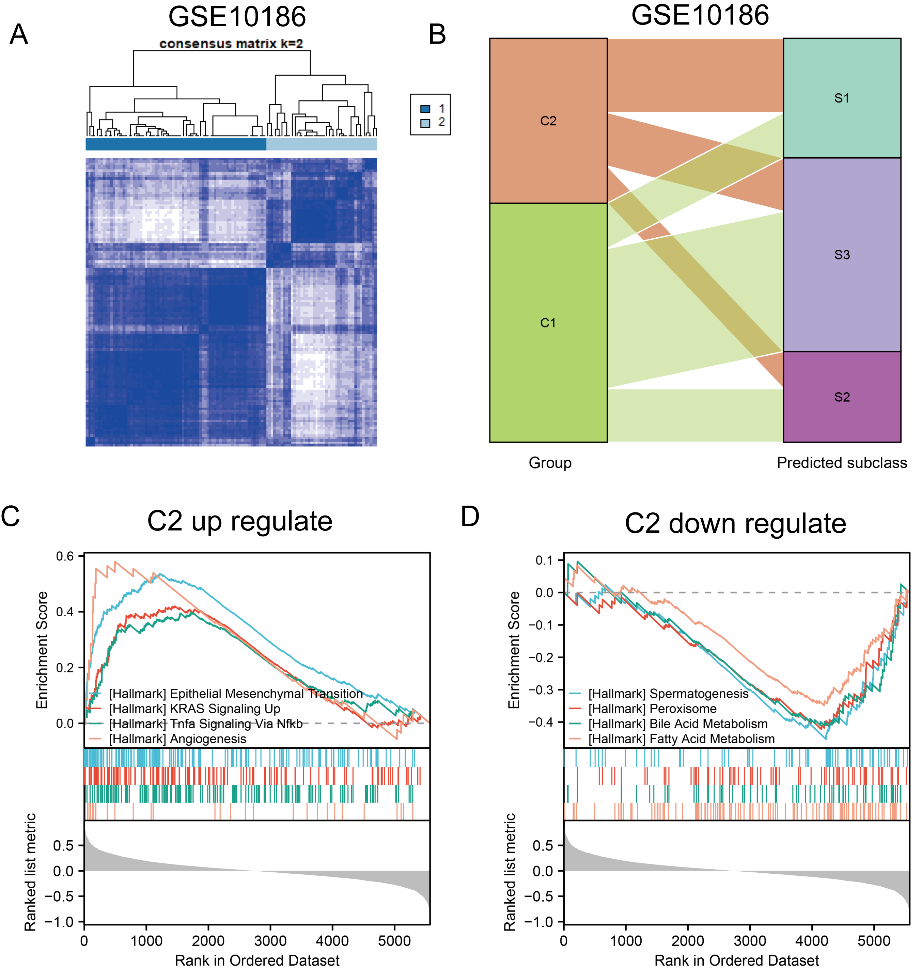


## Figure S4.

(A) The consensus score matrix of all samples when k = 2 based on GSE10186. (B) The Sankey diagram fully demonstrated the association between S1-3 subclass and subtypes attributes. (C-D) Up (C) and down (D) regulated HALLMARK pathways in C2. Different gene sets are represented by lines of different colors., and up-regulated genes are located on the left approaching the origin of the coordinates, while the down-regulated genes are on the right of the x-axis. Only gene sets with NOM p < 0.01 and FDR q < 0.06 were considered significant. The top 5 gene sets are displayed in the plot.


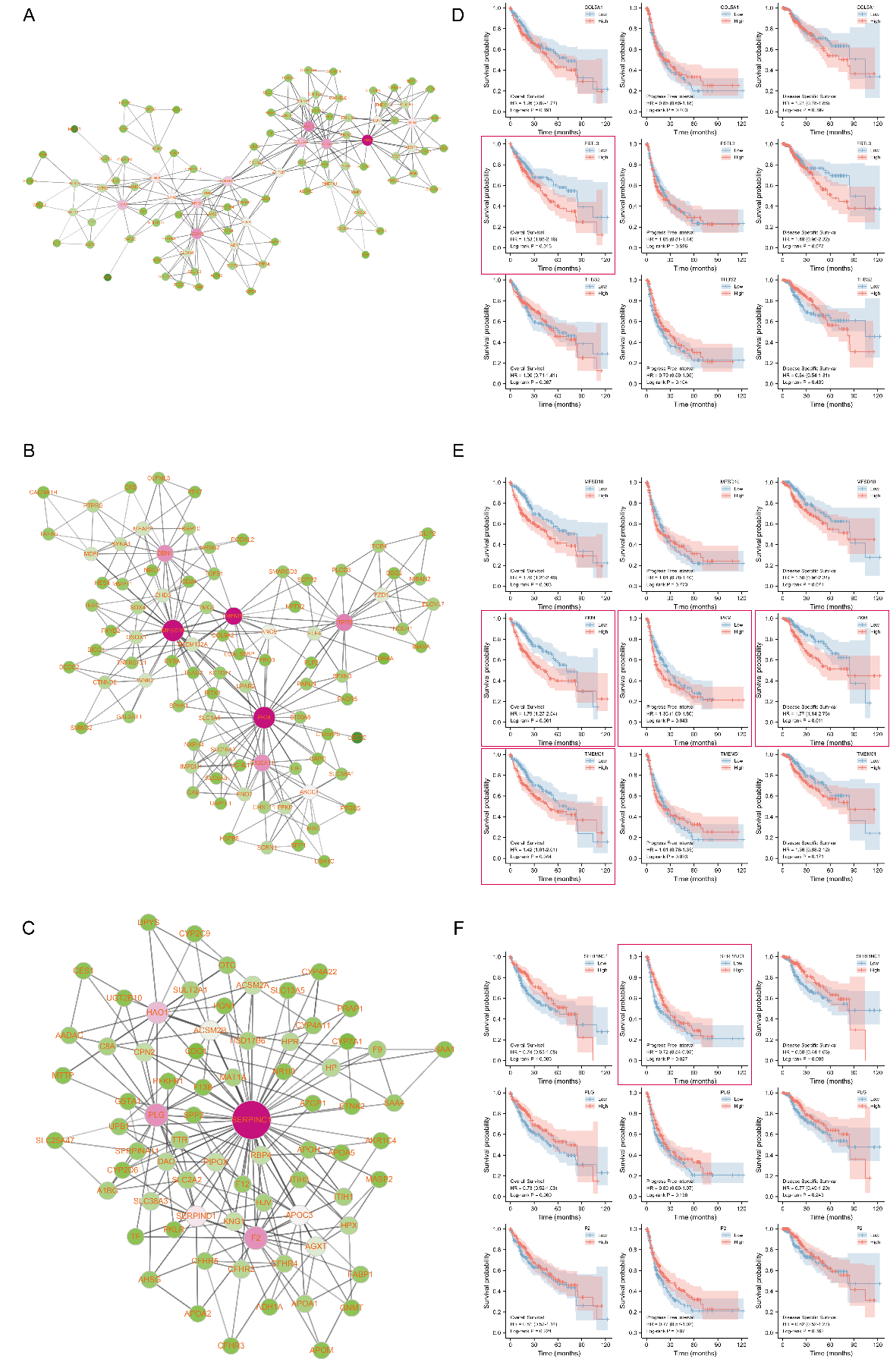


## Figure S5.

(A-C) The MEGENA network showing the top three gene module. Each color represents one module, and triangles represent key genes in the module. The darker the color of the node, the more important it is in the network, and the darker the color of the line, the greater the weight value between the nodes. (D-F) The top three key genes in each module and their prognostic ability in assessing the clinical outcome of HCC patients by using Kaplan-Meier Plotter.


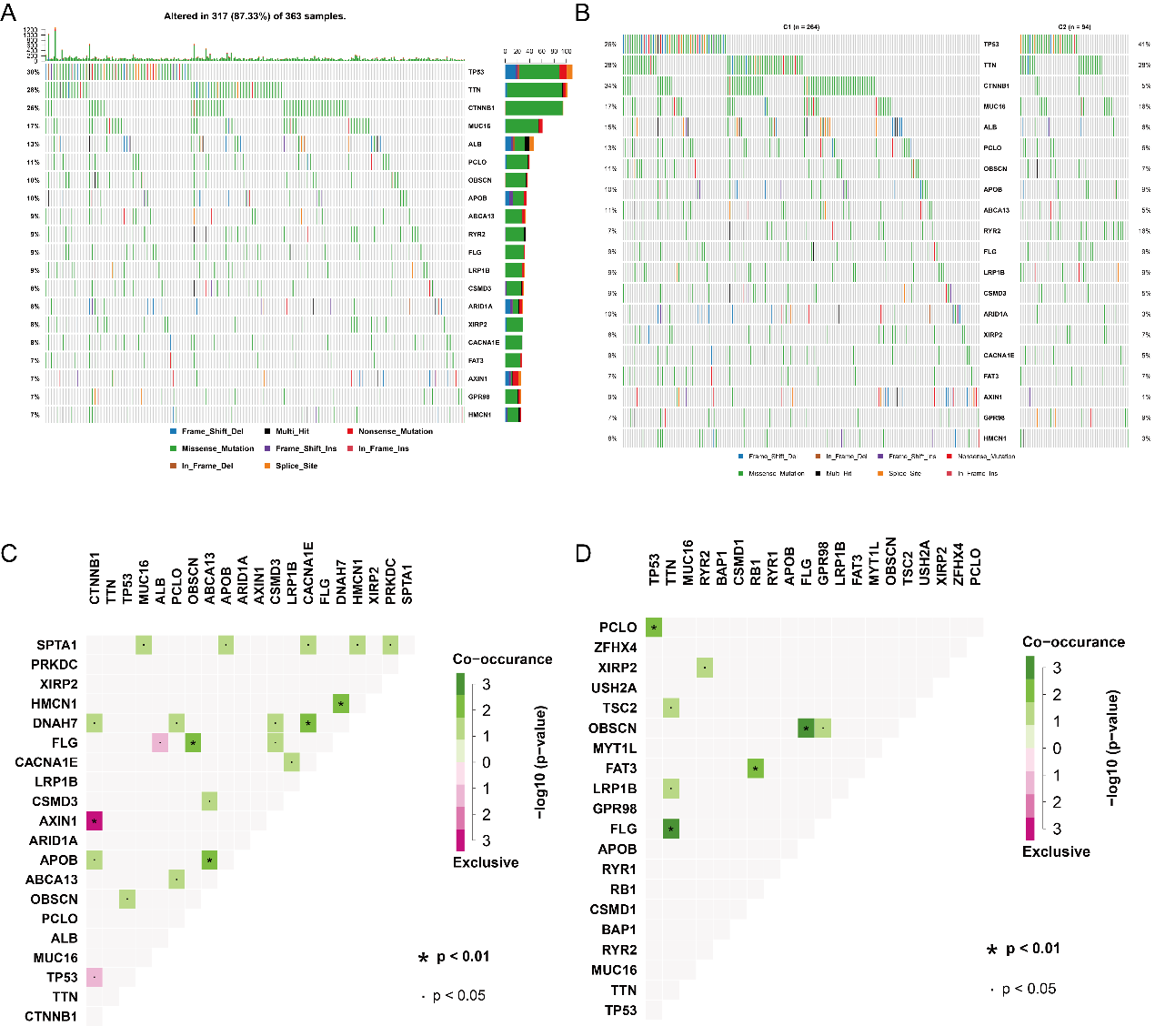


## Figure S6.

(A) Mutation annotation of the top 20 frequently mutated genes in the TCGA-LIHC cohort. (B) Comparison of the difference in mutation between C1 and C2 clusters. (C-D) Graphs showing mutually exclusive and co-occurrence of mutational events in C1 and C2, respectively.


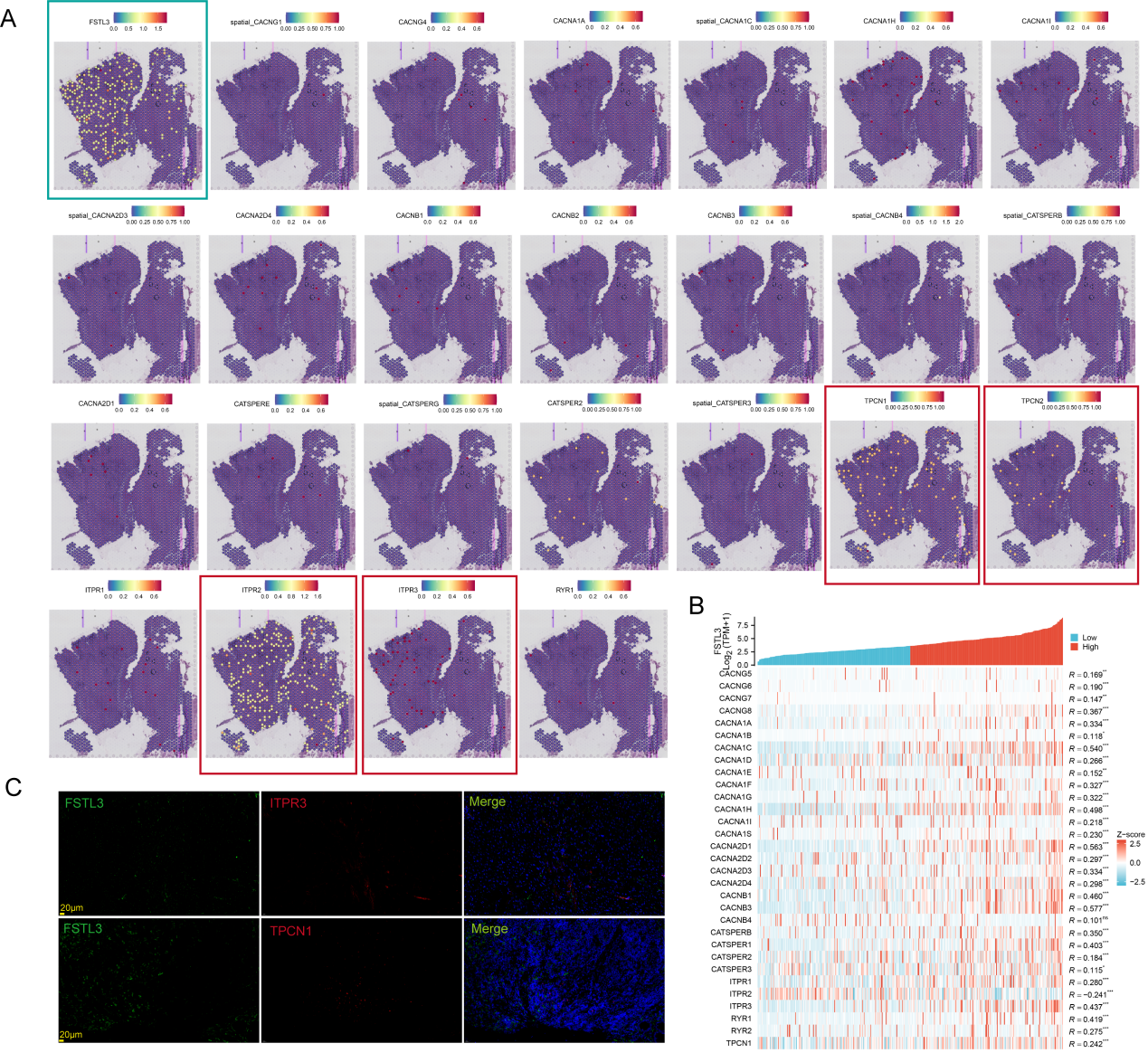


## Figure S7.

(A) Spatial transcription sections show the spatial expression of FSTL3 (green box) and calcium channel molecules in HCC tissue. The dot color represents the expression level. The redder the color, the higher the expression. Significantly up-regulated genes are highlighted within a red box (ITPR2, ITPR3, TPCN and TPCN2). (B) Spearman correlation between FSTL3 and calcium channel coding genes. Correlation coefficients and significance levels are displayed on the right side of the image. (C) Immunofluorescence (IF) staining images of FSTL3 (green) can be observed colocalized with ITPR3/TPCN1(red) in HCC specimen (blue, DAPI). Scale bars are labelled on the graph. *P < 0.05, **P < 0.01, ***P < 0.001


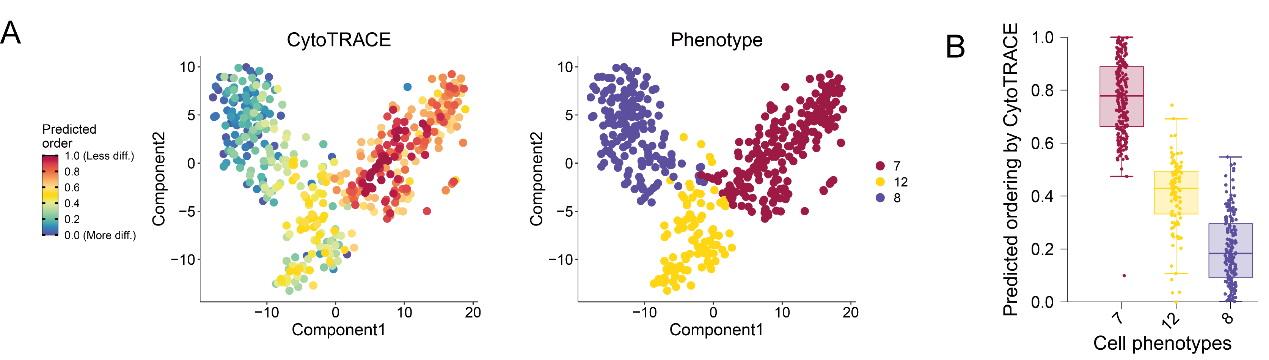


## Figure S8.

(A) tSNE demonstrating the degree of differentiation of each fibroblasts cluster assessed by CytoTRACE. (B) Box plot showing the differentiation score of each fibroblasts cluster.


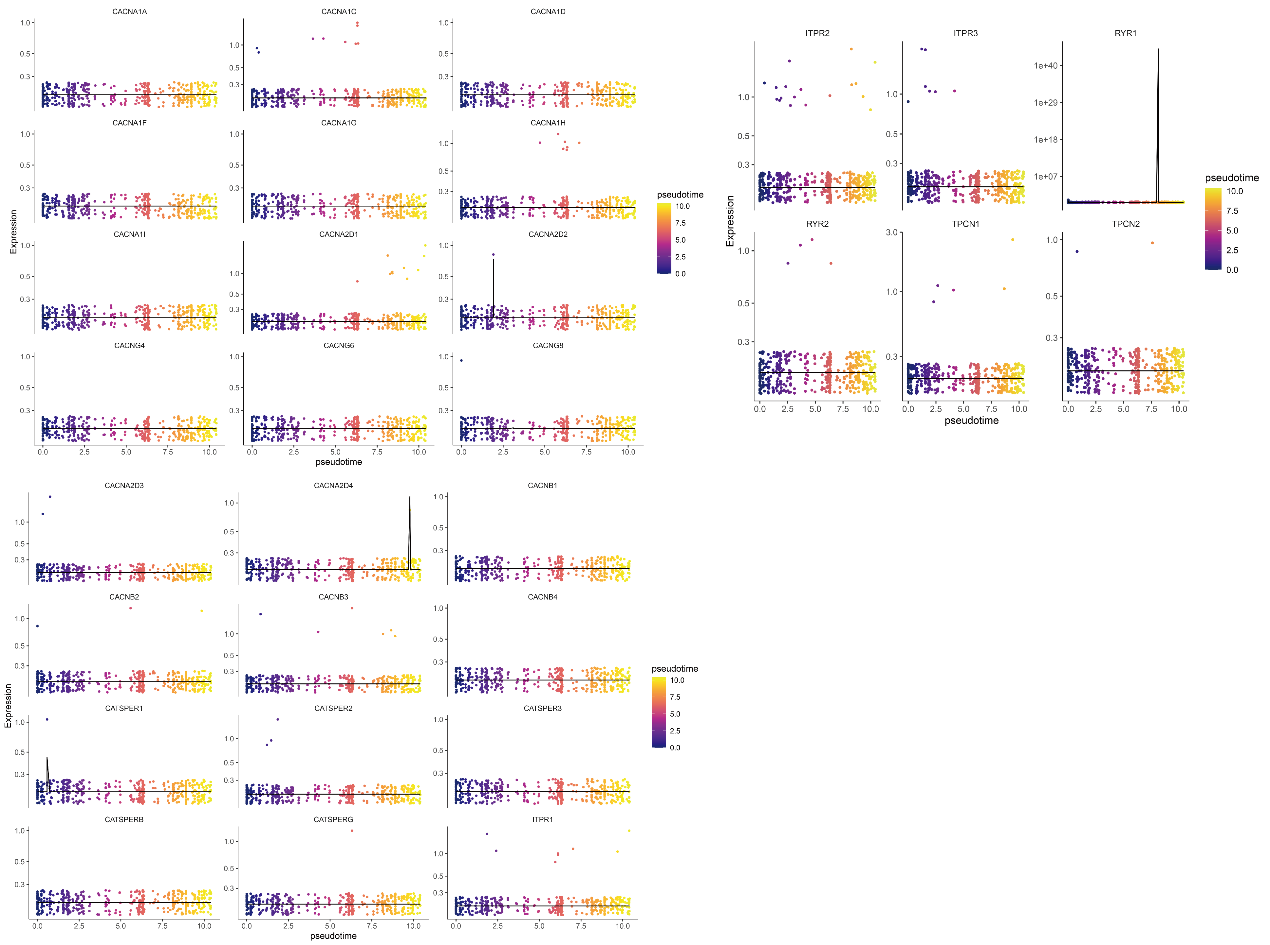


## Figure S9.

30 calcium channel molecules expression changes in pseudotime analysis for fibroblasts.


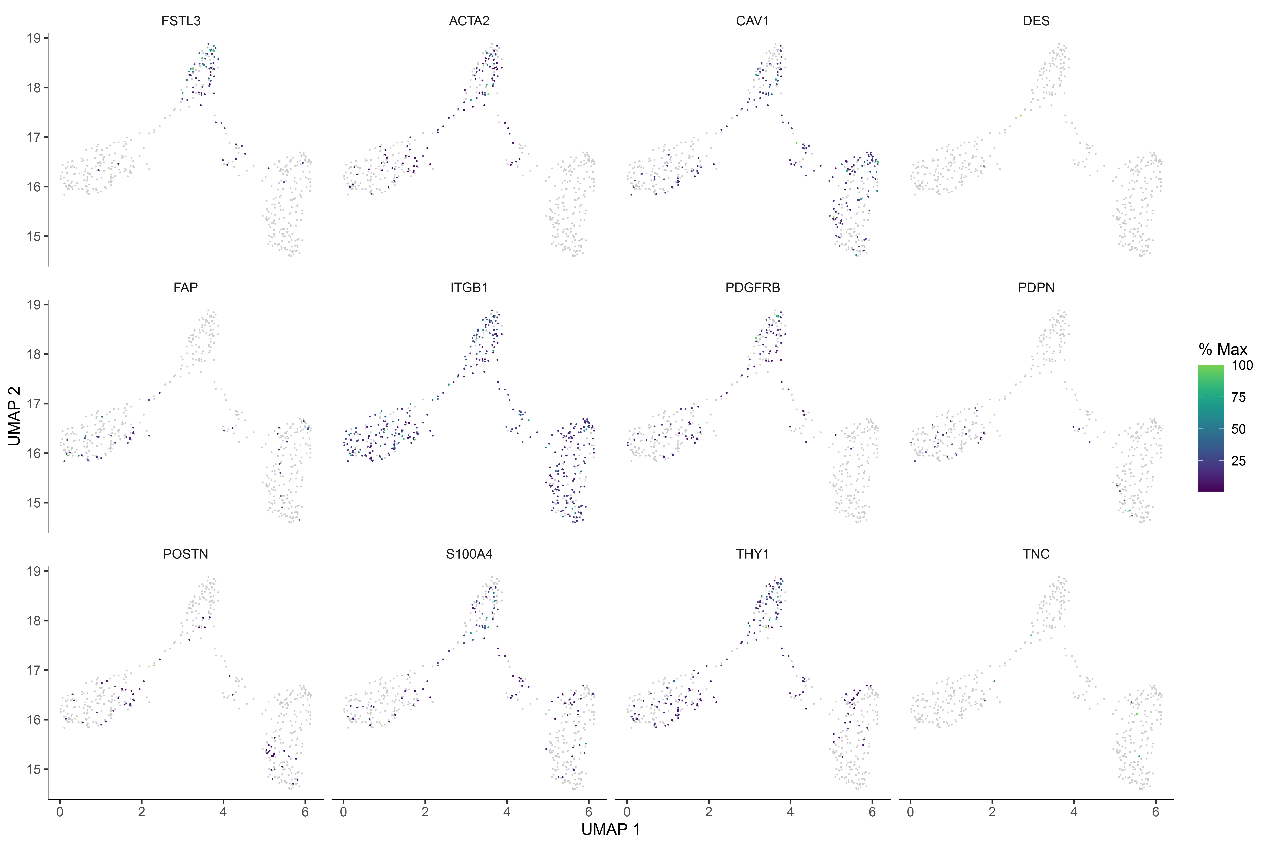


## Figure S10.

UMAP dimensionality reduction visualizes similarity of expression profiles of FSTL3 and 11 fibroblast activation markers.


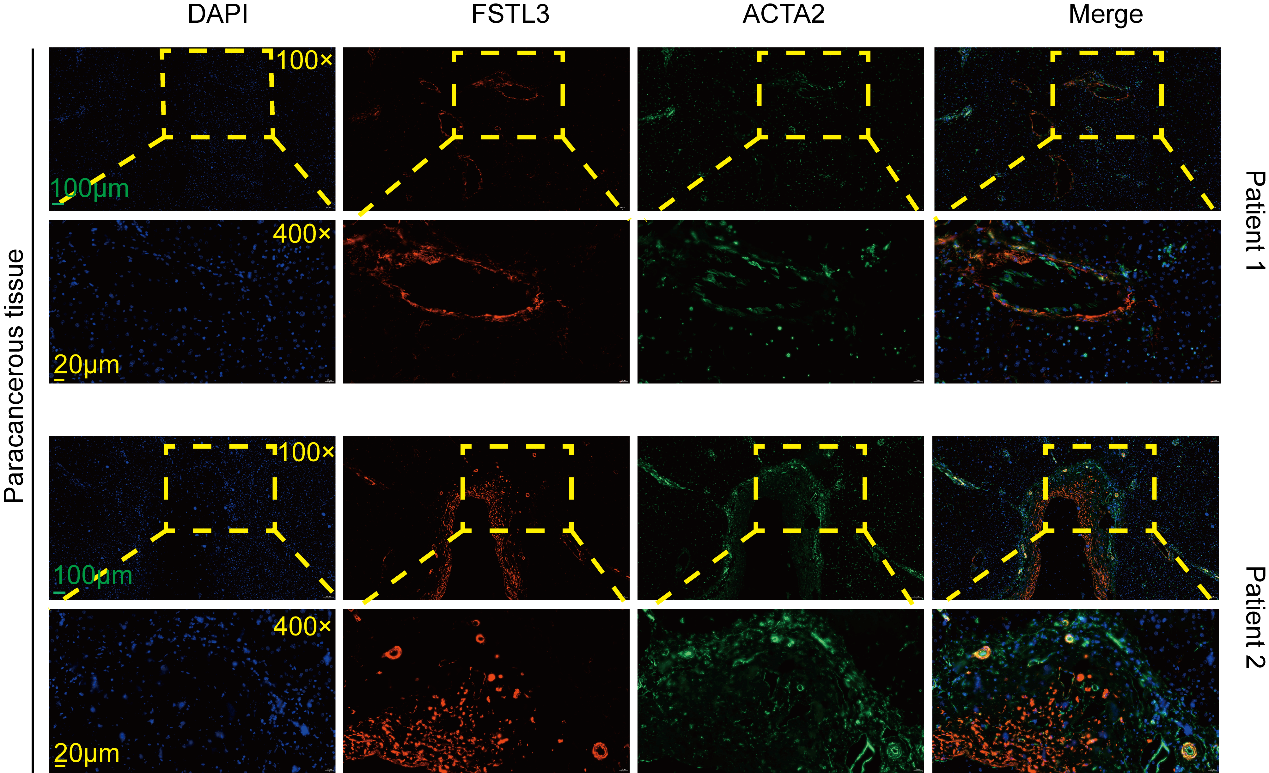


## Figure S11.

Double immunofluorescence staining images of FSTL3 and ACTA2 in the paracancerous tissue. The representative view of the staining of FSTL3 and ACTA2 is shown in the enlarged images view below. Scale bars, 100 and 20 μm (enlarged images). Nuclei (DAPI) in blue.


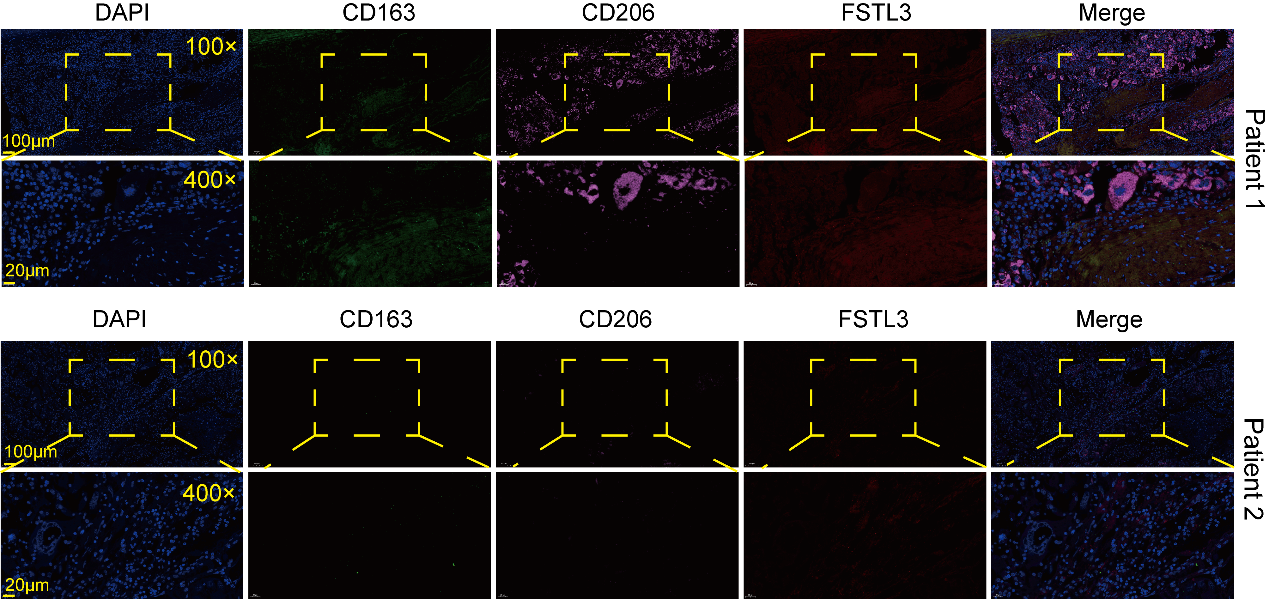


## Figure S12.

FSTL3, CD163, and CD206 triple immunofluorescence staining of HCC tissue. The triple staining is detailed in the enlarged images below. (Magnification, ×100, ×400, Scale bars, 100 and 20 μm (enlarged images). Nuclei (DAPI) stained blue.


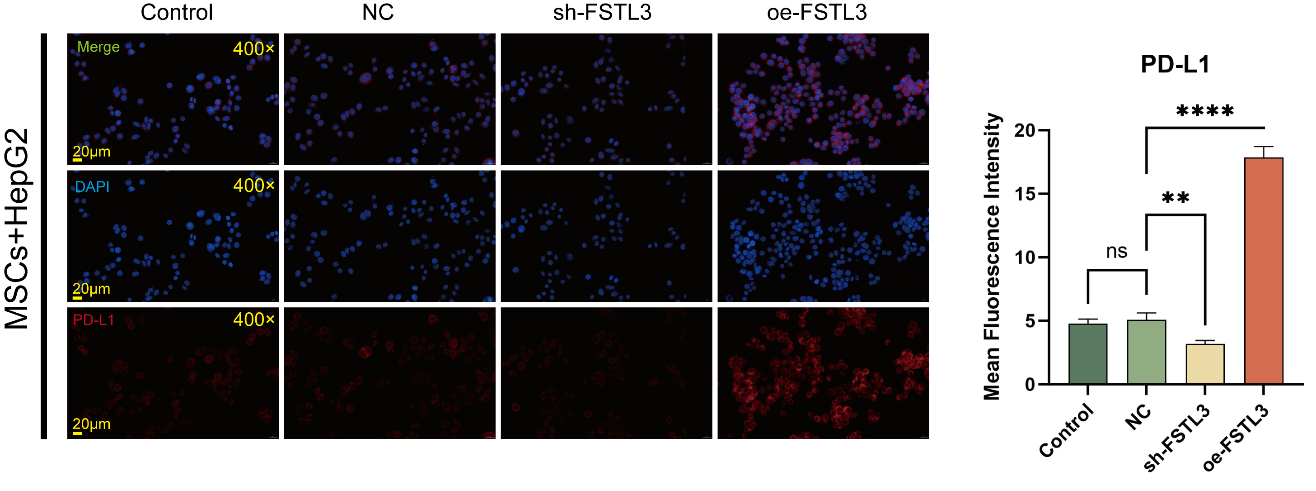


## Figure S13.

Establishment of a CAFs and HepG2 Co-culture System, PD-L1(red) immunofluorescence staining; nuclei was DAPI-stained (blue) (Magnification, ×400, scale bars = 20 μm) (n = 3 replicates). Immunofluorescence intensity (mean ± SEM) (n = 3 replicates). Data presented as means ± SEM, **p <0.01.


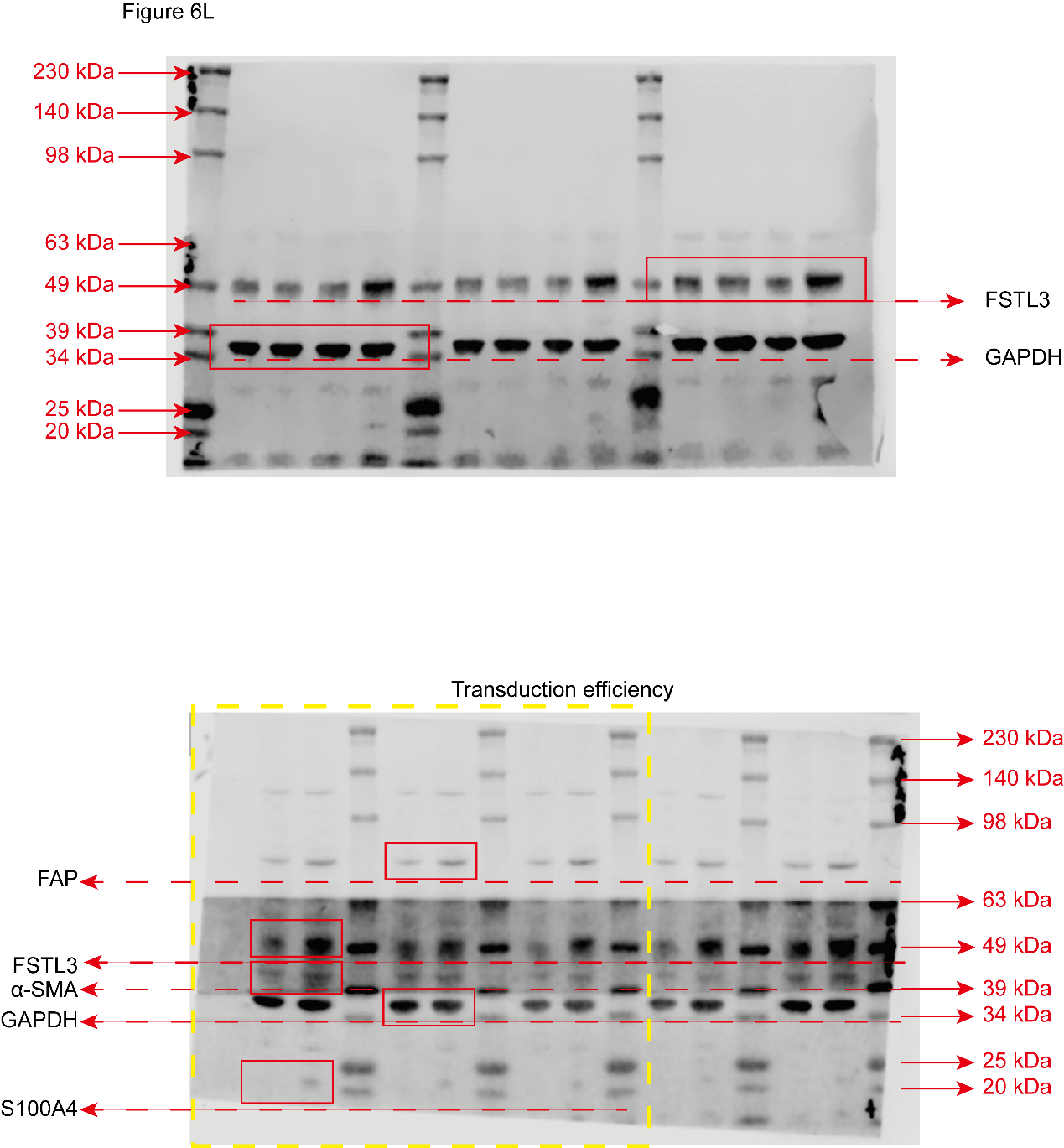


## Figure S14.

Uncut version of the blots. The data in solid red box were selected as representative images. The yellow box shows the results of the three replicates that were used for statistical purposes. Dashed arrows represent each protein bands.

# Supplementary Table

## Table S1 43 calcium channel-encoding genes from the HUGO

| HGNC ID (gene) | Approved symbol | Approved name | Chromosome |
| --- | --- | --- | --- |
| HGNC:1405 | CACNG1 | calcium voltage-gated channel auxiliary subunit gamma 1 | 17q24.2 |
| HGNC:1406 | CACNG2 | calcium voltage-gated channel auxiliary subunit gamma 2 | 22q12.3 |
| HGNC:1407 | CACNG3 | calcium voltage-gated channel auxiliary subunit gamma 3 | 16p12.1 |
| HGNC:1408 | CACNG4 | calcium voltage-gated channel auxiliary subunit gamma 4 | 17q24.2 |
| HGNC:1409 | CACNG5 | calcium voltage-gated channel auxiliary subunit gamma 5 | 17q24.2 |
| HGNC:13625 | CACNG6 | calcium voltage-gated channel auxiliary subunit gamma 6 | 19q13.42 |
| HGNC:13626 | CACNG7 | calcium voltage-gated channel auxiliary subunit gamma 7 | 19q13.42 |
| HGNC:13628 | CACNG8 | calcium voltage-gated channel auxiliary subunit gamma 8 | 19q13.42 |
| HGNC:1388 | CACNA1A | calcium voltage-gated channel subunit alpha1 A | 19p13.13 |
| HGNC:1389 | CACNA1B | calcium voltage-gated channel subunit alpha1 B | 9q34.3 |
| HGNC:1390 | CACNA1C | calcium voltage-gated channel subunit alpha1 C | 12p13.33 |
| HGNC:1391 | CACNA1D | calcium voltage-gated channel subunit alpha1 D | 3p21.1 |
| HGNC:1392 | CACNA1E | calcium voltage-gated channel subunit alpha1 E | 1q25.3 |
| HGNC:1393 | CACNA1F | calcium voltage-gated channel subunit alpha1 F | Xp11.23 |
| HGNC:1394 | CACNA1G | calcium voltage-gated channel subunit alpha1 G | 17q21.33 |
| HGNC:1395 | CACNA1H | calcium voltage-gated channel subunit alpha1 H | 16p13.3 |
| HGNC:1396 | CACNA1I | calcium voltage-gated channel subunit alpha1 I | 22q13.1 |
| HGNC:1397 | CACNA1S | calcium voltage-gated channel subunit alpha1 S | 1q32.1 |
| HGNC:1399 | CACNA2D1 | calcium voltage-gated channel auxiliary subunit alpha2delta 1 | 7q21.11 |
| HGNC:1400 | CACNA2D2 | calcium voltage-gated channel auxiliary subunit alpha2delta 2 | 3p21.31 |
| HGNC:15460 | CACNA2D3 | calcium voltage-gated channel auxiliary subunit alpha2delta 3 | 3p21.1-p14.3 |
| HGNC:20202 | CACNA2D4 | calcium voltage-gated channel auxiliary subunit alpha2delta 4 | 12p13.33 |
| HGNC:1401 | CACNB1 | calcium voltage-gated channel auxiliary subunit beta 1 | 17q12 |
| HGNC:1402 | CACNB2 | calcium voltage-gated channel auxiliary subunit beta 2 | 10p12 |
| HGNC:1403 | CACNB3 | calcium voltage-gated channel auxiliary subunit beta 3 | 12q13.12 |
| HGNC:1404 | CACNB4 | calcium voltage-gated channel auxiliary subunit beta 4 | 2q23.3 |
| HGNC:20500 | CATSPERB | cation channel sperm associated auxiliary subunit beta | 14q32.12 |
| HGNC:28598 | CATSPERD | cation channel sperm associated auxiliary subunit delta | 19p13.3 |
| HGNC:28491 | CATSPERE | catsper channel auxiliary subunit epsilon | 1q44 |
| HGNC:25243 | CATSPERG | cation channel sperm associated auxiliary subunit gamma | 19q13.2 |
| HGNC:19231 | CATSPERZ | catsper channel auxiliary subunit zeta | 11q13.1 |
| HGNC:17116 | CATSPER1 | cation channel sperm associated 1 | 11q13.1 |
| HGNC:18810 | CATSPER2 | cation channel sperm associated 2 | 15q15.3 |
| HGNC:20819 | CATSPER3 | cation channel sperm associated 3 | 5q31.1 |
| HGNC:23220 | CATSPER4 | cation channel sperm associated 4 | 1p36.11 |
| HGNC:6180 | ITPR1 | inositol 1,4,5-trisphosphate receptor type 1 | 3p26.1 |
| HGNC:6181 | ITPR2 | inositol 1,4,5-trisphosphate receptor type 2 | 12p11.23 |
| HGNC:6182 | ITPR3 | inositol 1,4,5-trisphosphate receptor type 3 | 6p21.31 |
| HGNC:10483 | RYR1 | ryanodine receptor 1 | 19q13.2 |
| HGNC:10484 | RYR2 | ryanodine receptor 2 | 1q43 |
| HGNC:10485 | RYR3 | ryanodine receptor 3 | 15q13.3-q14 |
| HGNC:18182 | TPCN1 | two pore segment channel 1 | 12q24.13 |
| HGNC:20820 | TPCN2 | two pore segment channel 2 | 11q13.3 |
